# Research on a CEF-YOLOv8n-Based Method for Small Object Detection in UAV Aerial Imagery

**DOI:** 10.3390/s26154818

**Published:** 2026-07-29

**Authors:** Yamei Zhang, Keding Yan

**Affiliations:** 1 School of Intelligent Science and Engineering, Xi’an Peihua University, Xi’an 710125, China; 150445@peihua.edu.cn; 2 School of Electronic and Information Engineering, Xi’an Technological University, Xi’an 710021, China

**Keywords:** UAV, target recognition, YOLOv8n, feature fusion, multi-scale

## Abstract

**Highlights:**

**What are the main findings?**

- A CEF-YOLOv8n model is proposed for small-object detection in UAV aerial images by introducing the CPF module into the YOLOv8n backbone to enhance feature extraction for low-resolution small objects.
- The FGFPN, FSFM, and LWSD modules are designed to improve multi-scale feature fusion, strengthen the interaction between shallow detail features and deep semantic features, and reduce redundant computation in the detection head.

**What are the implications of the main findings?**

- The CPF-based feature extraction scheme proposed in this paper can better retain the detailed features of small objects in UAV aerial images and alleviate feature loss arising from repeated convolution and downsampling operations.
- The combination of FGFPN, FSFM, and LWSD improves the representation of dense and multi-scale small objects while maintaining lightweight model design, enabling more accurate and efficient small-object detection in UAV aerial images.

**Abstract:**

To address the recognition challenges caused by the high proportion, low resolution, and significant multi-scale variations of small objects in UAV small-object detection tasks, a UAV small-object detection and recognition algorithm based on CEF-YOLOv8n is proposed. The proposed algorithm uses YOLOv8n as the baseline network and introduces a Partial Convolution-based Cross Partial Feature (CPF) module into the backbone network to enhance the local feature extraction capability for low-resolution small objects. In the neck network, the concept of feature focusing and diffusion is adopted to construct a Focusing Generalized Feature Pyramid Network (FGFPN). A Feature Semantic Fusion Module (FSFM) based on a cross-attention mechanism is designed to complementarily fuse shallow detail features with deep semantic features, thereby enhancing information interaction among objects at different scales. In addition, a Lightweight Weight-Sharing Detection Head (LWSD) is proposed to improve the computational efficiency and real-time performance of the model while maintaining detection accuracy. Publicly available datasets are used for network training and detection evaluation, and comparative experiments are conducted with other algorithms. The results show that the proposed detection and recognition algorithm achieves 37.6% and 22.6% in terms of mAP50 and mAP50-95, respectively, representing improvements of 3.4 and 2.6 percentage points over the original YOLOv8n. Meanwhile, the number of parameters and FLOPs are reduced from 3.2 M and 8.7 G to 2.5 M and 6.9 G, respectively.

## 1. Introduction

With the development of unmanned aerial vehicle (UAV) technology, UAVs have been widely applied in various industries, bringing great convenience to people’s lives and promoting the rapid development of the low-altitude economy. One of its important applications is to use UAVs as the platform and equip them with high-speed cameras, which can achieve the detection and realization of small ground targets. This is a currently widely used application. However, the detection equipment based on aerial vision platforms, due to the high flight altitude of the UAVs, results in the visual target images obtained by them showing weak features, which brings difficulties to the real-time monitoring of ground targets. Therefore, how to improve the small target recognition in the drone visual imaging system is a challenging problem.

At present, unmanned aerial vehicle (UAV) aerial target detection has gradually been applied in fields such as intelligent transportation [1], agricultural monitoring [2], power line inspection [3], environmental protection and disaster rescue [4]. Compared with conventional fixed surveillance equipment, UAVs offer several advantages, including high mobility, wide coverage, flexible imaging perspectives, and efficient information acquisition. They can rapidly obtain information on the distribution and status of objects over large-scale scenes, thereby providing an important basis for traffic scheduling, risk warning, resource management, and emergency decision-making [5]. Particularly in tasks such as road vehicle monitoring, personnel search, and inspection of complex areas, the accurate identification of small-scale objects in aerial images is essential for improving UAV environmental perception and intelligent operation capabilities [6]. Therefore, investigating UAV-based aerial small-object detection methods that simultaneously achieve high detection accuracy, computational efficiency, and scene adaptability is of great significance for advancing low-altitude intelligent monitoring systems and facilitating the practical deployment of object detection models on UAV platforms. However, the high proportion of small objects, low imaging resolution, and significant scale variations in UAV aerial images pose considerable challenges to object detection [7]. First, UAVs generally capture images from a high-altitude, top-down perspective, causing objects such as vehicles and pedestrians to occupy only a small number of pixels in the images. Consequently, distinguishable information such as edges, textures, and contours is limited. After repeated convolution and downsampling operations in the network, the originally weak feature responses may be further attenuated or even merged into the background information, thereby resulting in missed detections. Second, the contrast between low-resolution small objects and complex backgrounds, such as roads, buildings, and vegetation, is relatively low. Target features are easily affected by illumination changes, occlusion, and background noise, making it difficult for the network to accurately distinguish target regions from surrounding areas and increasing false detections and localization deviations. Meanwhile, continuous changes in UAV flight altitude, imaging angle, and target distance lead to significant scale differences among targets of the same category. Features at a single level are difficult to satisfy the classification and localization requirements of targets at different scales. Although shallow features contain rich spatial details, their semantic representation ability is limited; deep features contain stronger category semantics, but they tend to lose the positional information of small objects. Therefore, retaining fine-grained information of low-resolution small objects during feature extraction and enhancing effective interactions among features of different scales and levels are key issues that need to be addressed to improve the performance of small-object detection in UAV aerial images.

Based on a UAV aerial small-object image dataset, this paper constructs a CEF-YOLOv8n model with YOLOv8n as the baseline, forming a small-object detection and recognition method for UAV aerial images that balances detection accuracy and lightweight model design. The main contributions are as follows:

(1) A partial-convolution-based CPF module is introduced into the backbone network of YOLOv8n. Through feature splitting, partial convolution, and cross-layer feature concatenation, the module reduces redundant computation while enhancing feature extraction for low-resolution small objects.
(2) An FGFPN neck network based on the feature focusing-diffusion strategy is constructed. The FFStage module is used to enhance cross-scale feature aggregation, and DySample dynamic upsampling and SPDConv downsampling are combined to reduce the loss of detailed information of small objects during scale transformation.
(3) An FSFM feature semantic fusion module based on a cross-attention mechanism is designed to achieve complementary fusion of detailed features and category semantics, thereby improving the representation ability for dense small objects and multi-scale targets. In addition, an LWSD lightweight weight-sharing detection head is introduced. Through shared convolution, group normalization, and scale adjustment mechanisms, it reduces parameter redundancy and computational overhead in different detection branches while maintaining the classification and localization accuracy of small objects, thereby improving the inference efficiency and deployment adaptability of the model.

The remainder of this paper is organized as follows. Section 2 reviews the current research on small-object detection in UAV aerial images. Section 3 presents the proposed CEF-YOLOv8n method in detail, including the overall design, algorithmic process, and each improved module. Section 4 conducts comparative experiments, ablation experiments, and visualization analysis. Finally, Section 5 concludes the paper.

## 2. Related Work

Existing studies on small-object detection in UAV aerial scenes have primarily adopted deep learning-based object detection methods. According to differences in the detection pipeline, object detection algorithms can generally be divided into two-stage and one-stage detectors. Representative two-stage detectors include R-CNN [8], Fast R-CNN [9], Faster R-CNN [10], and Cascade R-CNN [11]. These methods typically first employ a region proposal network to generate candidate regions and then perform feature extraction, category classification, and bounding-box refinement on these regions. Although they generally achieve high detection accuracy, their complex processing pipelines and high computational costs make it difficult to satisfy the real-time detection requirements of UAV platforms. Representative one-stage detectors include SSD [12], RetinaNet [13], and the YOLO series [14]. These methods directly predict object categories and locations through end-to-end networks, offering advantages such as fast inference, relatively compact architectures, and convenient deployment. In particular, the YOLO series provides a favorable balance between detection accuracy and computational efficiency and has therefore become an important foundational framework for small-object detection in UAV aerial imagery.

To address the weak features, strong background interference, and significant scale variations of small objects in aerial images, researchers have conducted extensive studies on feature enhancement and multi-scale information interaction. Zhang et al. [15] proposed SFE-Faster R-CNN, which reduces information loss during feature propagation by fusing low-resolution features with strong semantic information and high-resolution features with weak semantic information. Battish et al. [16] combined multi-scale enhanced channel attention with dilated convolution, sub-pixel convolution, and an additional prediction head to improve object detection performance in aerial imagery. Wang et al. [17] proposed SCASNet, which enhances the utilization of contextual information surrounding objects through a spatial context-aware selection mechanism. Qiu et al. [18] designed the SECAConv module, which strengthens small-object feature representation through dynamic weight allocation and channel attention. Fan et al. [19] constructed the C2f-RE module using RepViTBlock and efficient multi-scale attention, thereby enhancing deep small-object feature extraction while reducing model complexity. Li et al. [20] proposed an occlusion estimation module and a two-stage progressive refinement strategy to improve the localization and recognition of occluded objects. Yang et al. [21] proposed AFPN, which alleviates feature degradation during cross-scale information propagation by progressively fusing features from adjacent levels. Amudhan et al. [22] constructed skip connections between shallow and deep features to introduce richer contextual information. Hu et al. [23] proposed FSENet, which suppresses interference from complex backgrounds through feature suppression and enhancement mechanisms. Liao et al. [24] constructed a bidirectional hybrid-convolution multi-level feature association and fusion branch that progressively injects local details into the detection feature pyramid. Du et al. [25] proposed CFPT, an upsampling-free feature pyramid network that reduces semantic discrepancies and information loss through cross-layer interaction. Zhang et al. [26] designed the SPD-Conv structure, which enlarges the receptive field while reducing the loss of fine-grained features of small objects.

Improved methods based on the YOLO series have also been widely applied to small-object detection in UAV aerial imagery. Bai et al. [27] increased the proportion of small objects through image slicing and combined a dynamic decoupled detection head with a bounding-box matching loss to improve detection robustness. Fu et al. [28] constructed the CFP_C2f module using partial convolution and combined it with a context aggregation module and normalized Wasserstein distance loss to improve feature extraction and localization regression. Wu et al. [29] introduced bi-level routing attention to filter irrelevant features in a dynamically sparse manner. Wang et al. [30] employed a global Contextual Transformer module and max-pooling operations to strengthen the extraction of small-object texture information under complex backgrounds, thereby improving detection accuracy. Zhao et al. [31] used a multi-attention Dynamic Head to enhance object detection performance in complex backgrounds and incorporated DenseNet to strengthen feature propagation. Chen et al. [32] added a small-object detection layer to YOLOv5 and combined it with C3Res2Block, a decoupled detection head, and Soft-NMS to improve the detection accuracy of densely distributed small objects. Zhou et al. [33] replaced the CSPDarknet backbone with MobileNet-V3 and employed Bi-FPN to enhance feature extraction and multi-scale feature fusion, thereby improving inference speed while reducing the number of parameters. Liu et al. [34] replaced the C3 module in YOLOv5 with a spatiotemporal interaction module and introduced recursive gated convolution into the PAN structure to improve small-object detection by exploiting shallow features and strengthening feature fusion. Zhang et al. [35] added a shallow feature extraction network to the prediction head of YOLOX and introduced the NAM attention mechanism into the backbone to suppress redundant feature representations, thereby alleviating missed and false detections of small-scale objects in high-altitude aerial images. Mao et al. [36] proposed MSA-YOLO, which employs a multi-scale feature extraction module to capture feature information at different scales and improve object detection performance in UAV imagery.

The above studies have made considerable progress in small-object feature enhancement, multi-scale information fusion, attention modeling, detection-head optimization, and model lightweight design. However, under the conditions of a high proportion of small objects, low resolution, and significant scale variations in UAV aerial images, problems remain, including the easy loss of local details during multiple convolutions and scale transformations, large semantic differences between deep and shallow features, and repeated computation in detection branches. Some methods improve accuracy by adding detection layers or introducing complex attention structures, but they may also increase the number of parameters and computational cost, which is unfavorable for deployment on resource-constrained platforms. Therefore, based on YOLOv8n, this paper constructs CEF-YOLOv8n for small-object detection in UAV aerial images. By introducing the CPF module, FGFPN neck network, FSFM module, and LWSD lightweight weight-sharing detection head, the proposed model reduces parameter redundancy in multi-scale detection branches, thereby improving its detection ability for low-resolution, multi-scale, and densely distributed small objects while maintaining computational efficiency.

## 3. Small Object Detection and Recognition Method for UAV Aerial Images Based on CEF-YOLOv8n

### 3.1. Overall Design

**Figure 1:**
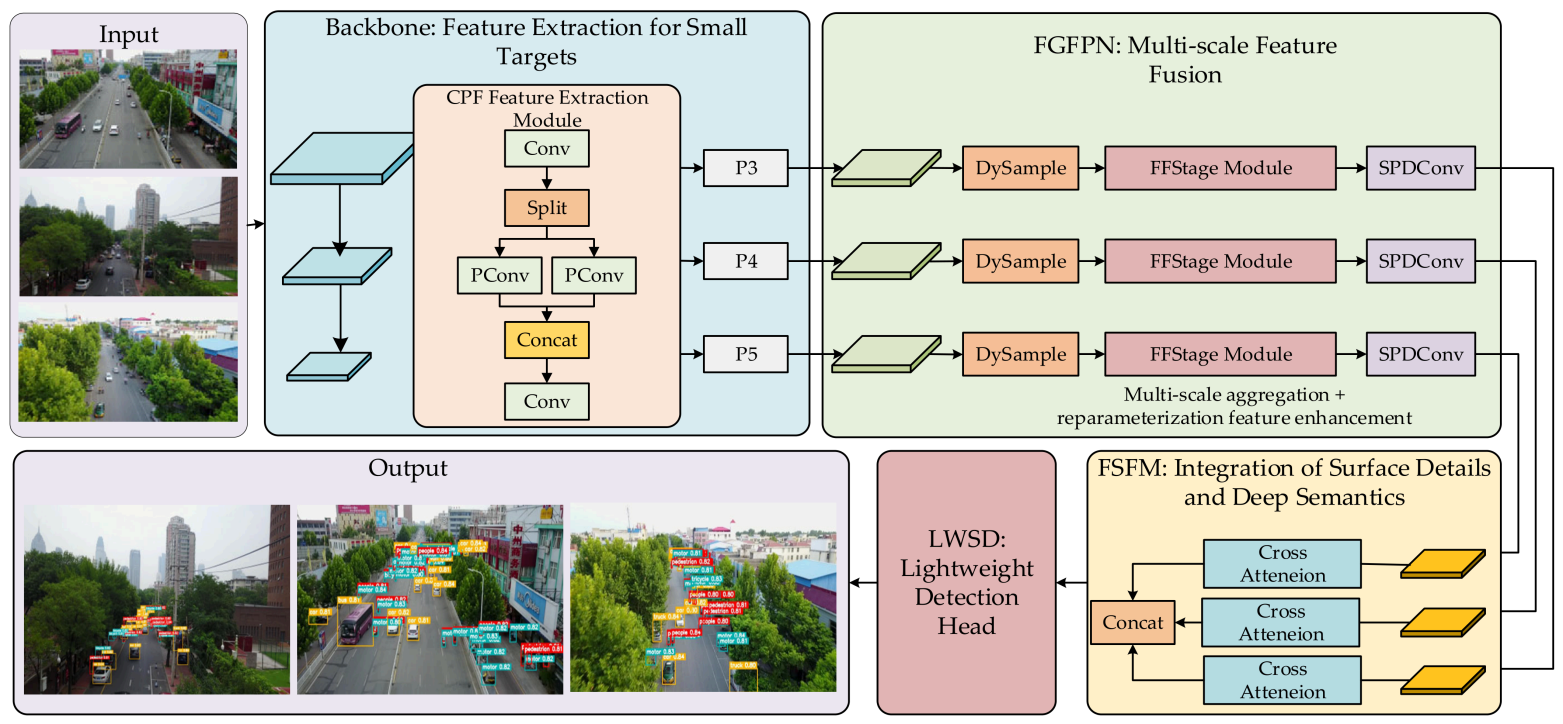
Overall design framework.

To address the recognition difficulties caused by the high proportion, low resolution, and significant scale variations of small objects in UAV aerial images, this paper proposes a UAV aerial small-object detection model, termed CEF-YOLOv8n, based on YOLOv8n. The proposed model introduces CPF, FGFPN, FSFM, and LWSD into the backbone network, neck network, feature fusion stage, and detection head, respectively. By preserving the local details of small objects, enhancing the interaction among features at different scales, supplementing semantic information between shallow and deep layers, and reducing computational redundancy at the detection end, the model improves its detection capability for small objects in UAV aerial imagery. The overall design framework of the model is shown in Figure 1.

First, the UAV aerial image is fed into the backbone network, where some of the original C2f modules are replaced with CPF modules. To address the high proportion, low resolution, and limited effective pixel information of small objects in aerial images, CPF splits the input features along the channel dimension. Spatial convolution is performed only on a portion of the channels, while the remaining channels retain more of the original information. The features are then fused through cross-layer feature concatenation. While reducing redundant computation, this structure preserves the edge, texture, and weak-response information of low-resolution small objects, thereby producing P3-, P4-, and P5-level features containing richer small-object details and providing effective inputs for subsequent multi-scale feature fusion.

Second, to improve the model’s representation capability for objects at different scales, the P3-, P4-, and P5-level features output by CPF are fed into FGFPN. FGFPN employs FFStage to aggregate features from different levels, enabling the local details extracted by the backbone network to be propagated across features of different scales. During feature-scale transformation, DySample and SPDConv are used for upsampling and downsampling, respectively. DySample dynamically adjusts the sampling positions according to the content of the input features, whereas SPDConv rearranges spatial information into the channel dimension, thereby reducing the loss of positional, edge, and texture information of small objects during scale transformation. Through these operations, the small-object details preserved by CPF can further participate in information interaction among features at different scales, allowing the model to better adapt to the significant scale variations of objects in UAV aerial imagery.

Subsequently, the P3-, P4-, and P5-level features output by the backbone network and the corresponding-scale features output by FGFPN are jointly fed into FSFM. The multi-level features produced by the backbone network retain spatial location, edge, and texture information at different resolutions, whereas the features output by FGFPN contain richer contextual information and stronger semantic representations after cross-scale aggregation. FSFM employs a cross-attention mechanism to establish bidirectional interactions between the two groups of multi-scale features, allowing the object details retained by the backbone network to complement the semantic information aggregated by FGFPN and generating fused features that contain both spatial details and category semantics. This structure not only continues to utilize the small-object information preserved by CPF in the backbone network but also incorporates the enhanced multi-scale features generated by FGFPN, thereby improving the model’s classification and localization capabilities for low-resolution, scale-varying, and densely distributed small objects.

Finally, considering that the feature-enhancement and multi-scale fusion structures may introduce additional computational overhead, the three-scale fused features output by FSFM are fed into the LWSD lightweight weight-sharing detection head. LWSD shares convolutional weights across detection branches of different scales to reduce repeated feature transformations and redundant parameter configurations, while group normalization is employed to improve the stability of feature processing. To accommodate the differences among features at different scales, the regression branch uses Scale layers to adaptively calibrate the prediction results. In this way, the parameter redundancy and computational complexity of the detection head are reduced while preserving the classification and localization capabilities for objects at different scales.

### 3.2. Construction of the CEF-YOLOv8n Network Model

**Figure 2:**
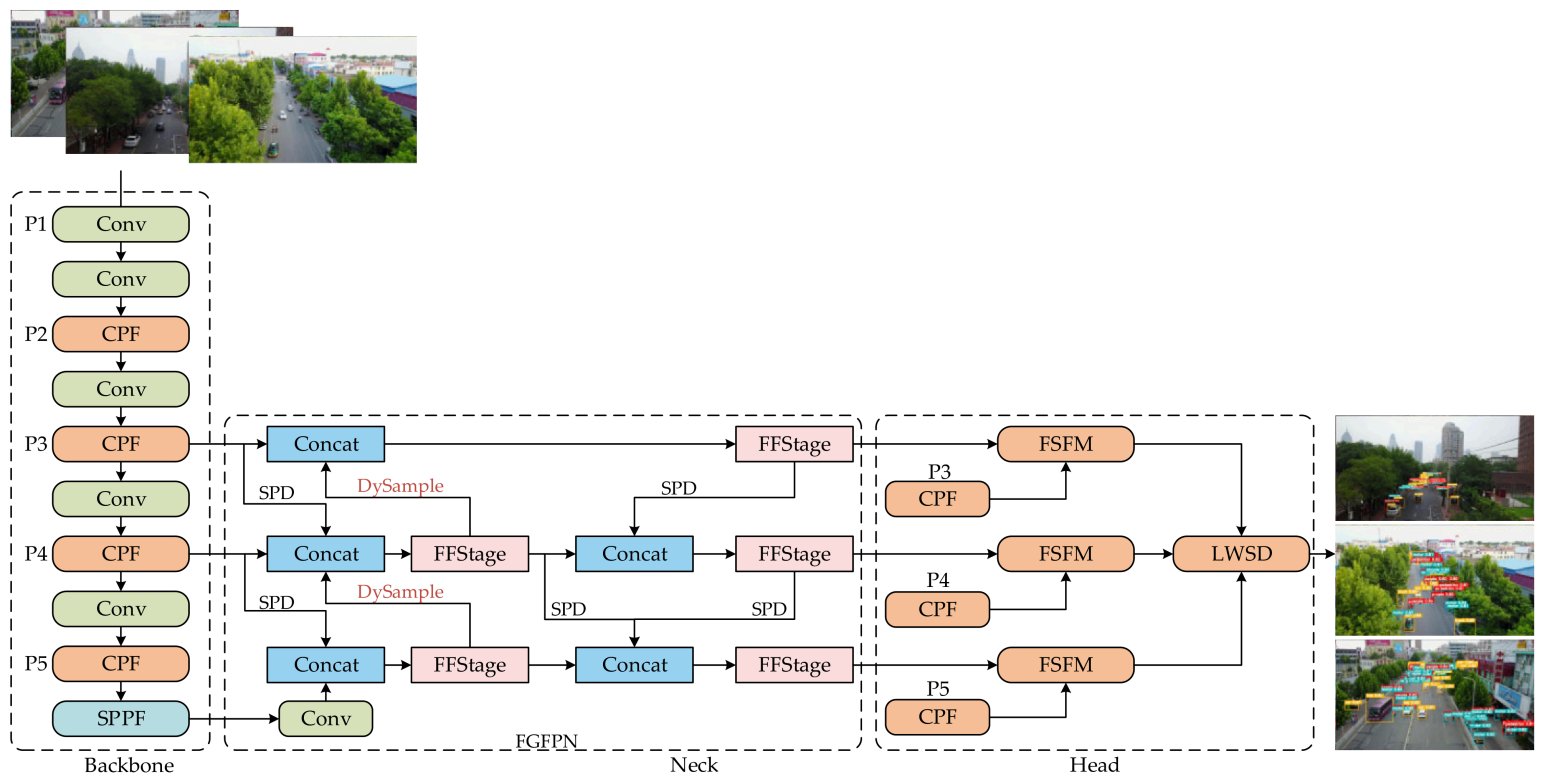
Structure of the CEF-YOLOv8n Model.

To address the problems of the high proportion of small objects, low resolution, and significant multi-scale variations in UAV aerial images, this paper constructs a CFL-YOLOv8n model for small-object detection in UAV aerial images based on the YOLOv8n framework. In the backbone network, the model introduces a partial-convolution-based CPF module to enhance the extraction of local detailed features from low-resolution small objects. In the neck network, an FGFPN structure based on the feature focusing-diffusion strategy is constructed to strengthen information interaction among features at different scales. In the detection head, the FSFM feature semantic fusion module and the LWSD lightweight weight-sharing detection head are combined to improve small-object detection accuracy while maintaining model inference efficiency. The overall structure of CEF-YOLOv8n is shown in Figure 2.

In the backbone network, a partial-convolution-based CPF module is introduced to replace some of the original C2f modules, so as to reduce redundant spatial computation and enhance the extraction of edge, texture, and weak-response features of low-resolution small objects. In the neck network, FGFPN is adopted to replace the original feature fusion structure. Multi-scale feature focusing and diffusion are achieved through FFStage, while DySample and SPDConv are used to replace traditional upsampling and downsampling operations, respectively, thereby reducing the loss of small-object details during scale transformation. The FSFM module is introduced to fuse shallow spatial details with deep semantic information. At the detection end, LWSD is used to replace the original decoupled detection head. Through multi-scale weight sharing and scale calibration, parameter redundancy is reduced, thereby improving the classification and localization accuracy of small objects while maintaining the computational efficiency of the model.

#### 3.2.1. CPF Module Based on Partial Convolution

**Figure 3:**
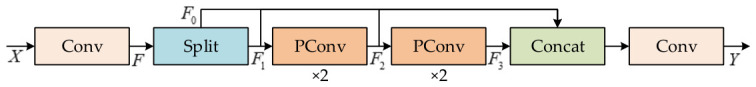
Structure of CPF Module.

In UAV aerial small-object detection, images are typically characterized by a high proportion of small objects, low target resolution, and substantial scale variation. Although the C2f modules in the original YOLOv8n backbone exhibit favorable lightweight characteristics, their convolution operations are generally applied to all channels. While extracting local features, these operations may also introduce redundant background information, making the detailed features of small objects susceptible to interference from complex backgrounds, while incurring additional computational overhead as the network deepens. To address these issues, a CPF module based on partial convolution is introduced into the YOLOv8n backbone to enhance the feature representation of low-resolution small objects in UAV aerial images. The structure of the CPF module is shown in Figure 3. This module applies spatial convolution to only a subset of the channels, while the remaining channels participate in subsequent feature fusion through an approximate identity mapping. Consequently, it reduces redundant computation and model complexity while preserving more original detail information from small objects, thereby enhancing the feature extraction capability of the model.

Specifically, the CPF module first performs a conventional convolution operation on the input feature tensor to complete initial feature mapping, and then splits the obtained features along the channel dimension. Subsequently, part of the split feature tensor is fed into the partial convolution group to further extract high-level semantic features. During this process, the module introduces two shortcut connections to enhance gradient flow and alleviate gradient attenuation during deep feature propagation, thereby accelerating network convergence. Finally, the feature tensors obtained from different branches are concatenated and fused through a convolution operation to obtain the output feature tensor of the CPF module. The feature extraction process of the CPF module can be expressed by Equations (1)–(4):

(1) $$F={\mathrm{Conv}}_{1}(X),$$

(2) $$\mathrm{Split}(F)=(F_{0},F_{1}),$$

(3) $$F_{2}={\mathrm{PConv}}_{2}({\mathrm{PConv}}_{1}(F_{1}))\;F_{3}={\mathrm{PConv}}_{4}({\mathrm{PConv}}_{3}(F_{2})),$$

(4) $$Y={\mathrm{Conv}}_{2}(\mathrm{Cat}(F_{0},F_{1},F_{2},F_{3})).$$

#### 3.2.2. FGFPN Feature Pyramid Network

The neck network of the original YOLOv8n mainly realizes the fusion of features at different levels through feature concatenation, upsampling, and downsampling operations. This fusion strategy has a relatively straightforward structure. Although it can achieve basic multi-scale information transmission, it is difficult for this approach to fully model the complex correlations among features at different scales, and some shallow detail information and deep semantic information cannot be effectively utilized during the fusion process. For UAV aerial images, targets usually have characteristics such as low pixel proportion, significant scale variations, and high-density distribution. If multi-scale feature fusion is insufficient, missed detections and false detections are likely to occur for distant small targets and targets in dense regions. To enhance the model’s ability to fuse small-target features at different scales, this paper constructs the FFStage feature focusing module and, on this basis, proposes the FGFPN neck network to improve the quality of multi-scale feature fusion and enhance the model’s detection ability for small targets in UAV aerial images.

**Figure 4:**
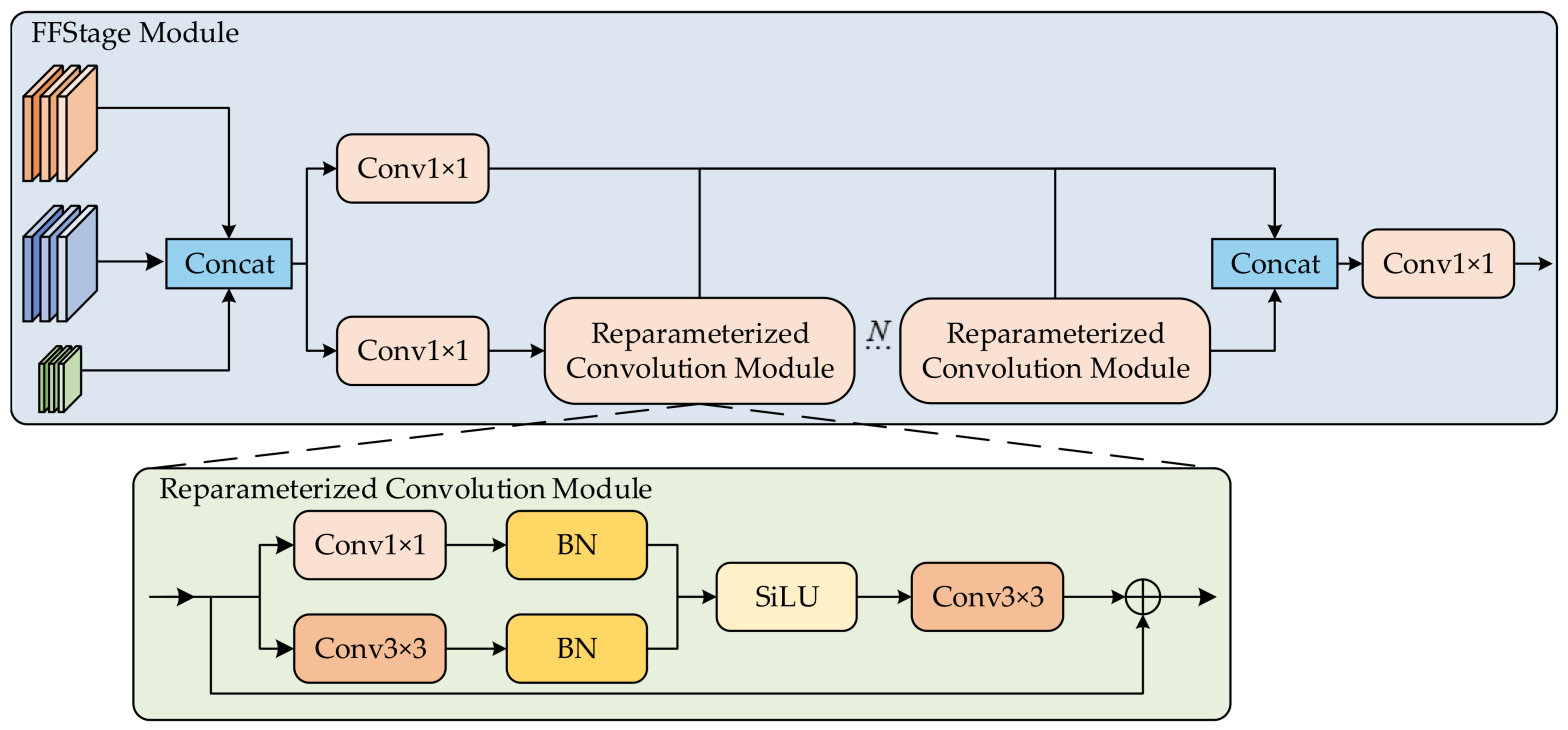
Structure of the FFStage Module.

Compared with the original YOLOv8n neck network, FGFPN introduces the FFStage module during feature fusion, as shown in Figure 4. This module can receive feature inputs from three scales and aggregate and represent features at different levels through an internal re-parameterized convolution structure. By extracting cross-scale contextual information through parallel convolution groups, FFStage reduces redundant computation while maintaining the feature fusion effect, thereby lowering model complexity. As a result, FGFPN can make fuller use of shallow small-target detail information and deep semantic information, improving the model’s feature representation capability in high-density and multi-scale aerial scenes. The re-parameterized convolution module first extracts local spatial information and channel mapping information through a 3 × 3 convolution branch and a 1 × 1 convolution branch, respectively, and then fuses the two feature streams. The calculation process is shown in Equations (5) and (6).

(5) $$G_{i}=\mathrm{SiLU}\left[\mathrm{BN}\left({\mathrm{Conv}}_{3\times 3}^{i}(Z_{i-1})\right)+\mathrm{BN}\left({\mathrm{Conv}}_{1\times 1}^{i}(Z_{i-1})\right)\right],$$

(6) $$Z_{i}=Z_{i-1}+{\mathrm{Conv}}_{3\times 3}^{i}(G_{i}),\;i=1,2,\ldots ,N,$$

where $Z_{i-1}$ and $Z_{i}$ denote the input and output of the (*i*)-th re-parameterized convolution module, respectively; $G_{i}$ denotes the intermediate feature obtained after fusing the two parallel convolution branches; BN(⋅) denotes batch normalization; SiLU(⋅) denotes the SiLU activation function; + denotes element-wise addition; and N denotes the number of stacked re-parameterized convolution modules.

The multi-scale feature fusion process of the FFStage module includes scale alignment, channel concatenation, construction of feature preservation branches, and re-parameterized branch processing. The specific calculation process is shown in Equations (7)–(10):

(7) $$U=\mathrm{Cat}\left(A_{1}(X_{1}),A_{2}(X_{2}),\ldots ,A_{m}(X_{m})\right),$$

(8) $$B={\mathrm{Conv}}_{1\times 1}(U),$$

(9) $$Z_{0}={\mathrm{Conv}}_{1\times 1}(U),\;m\leq 3,$$

(10) $$Y={\mathrm{Conv}}_{1\times 1}\left[\mathrm{Cat}\left(B,Z_{1},Z_{2},\ldots ,Z_{N}\right)\right],$$

where $A_{m}$ denotes the size alignment operation for the s-th scale feature, which can be implemented by upsampling, downsampling, or identity mapping; U denotes the initial fused feature obtained after features at different scales are concatenated; B denotes the output of the upper feature-preservation branch; $Z_{0}$ denotes the initial input of the lower re-parameterized convolution branch; $Z_{i}$ denotes the output of the i-th re-parameterized convolution module; Cat(⋅) denotes concatenation along the channel dimension; and Y denotes the final output of the FFStage module.

Although the feature diffusion strategy of FGFPN enhances information interaction among multi-scale features, the additional upsampling and downsampling operations may lead to the loss of local details of small targets during scale transformation and increase the computational cost of the model. Therefore, this paper adopts the lightweight dynamic upsampling method DySample to replace traditional upsampling operations. By adaptively adjusting sampling positions according to feature content, DySample improves the restoration quality of details for low-resolution small targets. Meanwhile, SPDConv is used for feature downsampling, which rearranges spatial–dimensional information into the channel dimension, thereby reducing the loss of edge, texture, and weak-response features caused by strided convolution and pooling. The combination of these two methods can reduce the number of model parameters and computational complexity while improving the retention of fine-grained features during scale transformation, thus improving the fusion and detection performance of multi-scale small targets.

By introducing the FFStage feature focusing module and the multi-level feature diffusion strategy, the FGFPN neck network enhances information transmission and interaction among features at different levels, enabling feature maps at different scales to obtain richer local details and contextual semantic information. This structure can effectively improve the model’s multi-scale feature fusion capability, enhance the representation of low-resolution targets, targets with significant scale variations, and densely distributed targets, and provide more sufficient feature support for subsequent classification and localization of small targets in UAV aerial images.

#### 3.2.3. FSFM Feature Semantic Fusion Module

**Figure 5:**
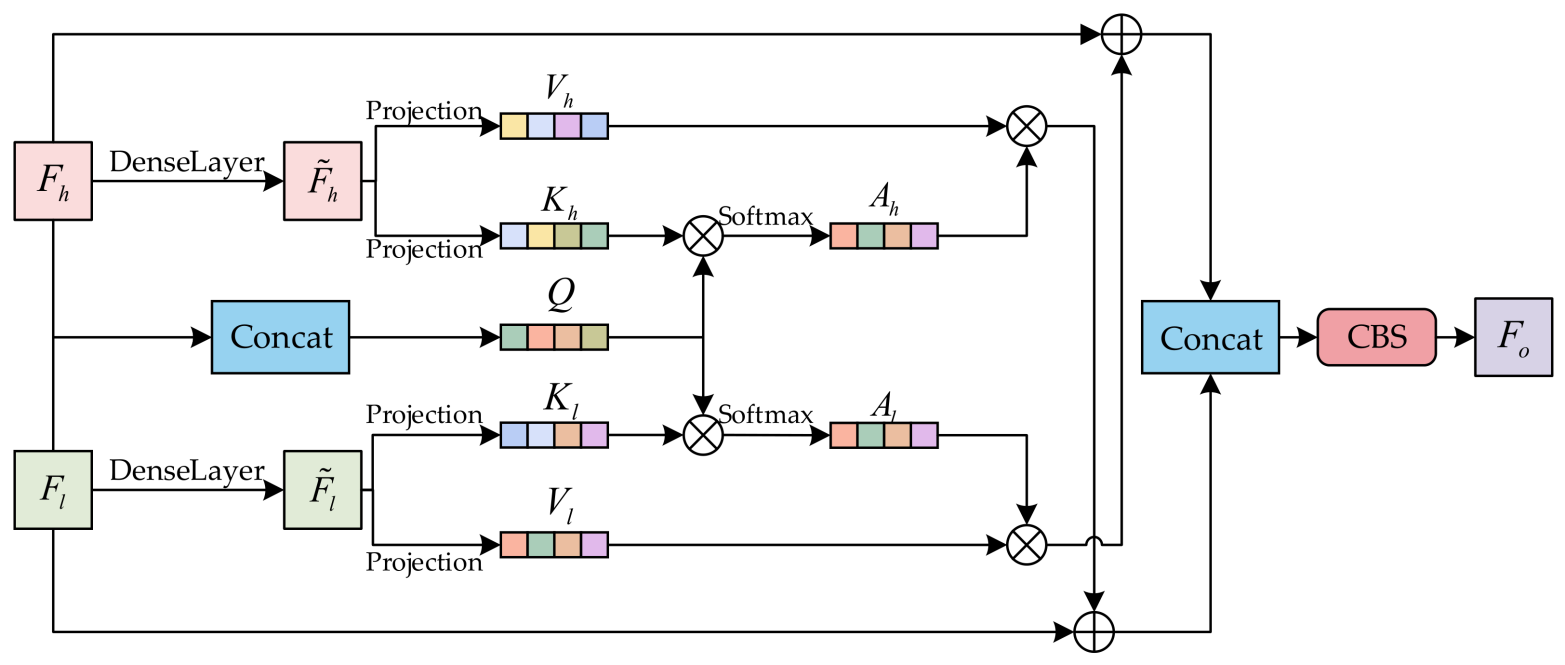
Schematic Diagram of the FSFM Feature-Semantic Fusion Module.

The FGFPN neck network can enhance information interaction among features at different scales, enabling the output features to contain richer multi-scale semantic information. However, as the network depth increases and multiple scale transformations are performed, the edge, contour, and positional information of small targets in aerial images is easily weakened. This problem is particularly evident in scenarios where targets are small and densely distributed. The loss of fine-grained features may make adjacent targets difficult to distinguish, thereby affecting classification and localization accuracy. Considering that shallow features from the backbone network contain rich spatial details but have limited semantic representation capability, while deep features from the neck network contain stronger category semantics and contextual information but have limited ability to perceive the local structures of small targets, this paper designs an FSFM feature-semantic fusion module based on a cross-attention mechanism. Through dense feature enhancement, cross-branch correlation modeling, and bidirectional information complementation, this module adaptively fuses shallow detail features and deep semantic features, thereby generating fused features that contain both spatial details and high-level semantics. These fused features provide more sufficient feature support for the classification and bounding-box localization of densely distributed small targets in aerial images. The structure of the module is shown in Figure 5.

FSFM takes the P3-, P4-, and P5-level features output by the backbone network and the corresponding-scale features output by FGFPN as inputs. First, Dense Layers are employed to enhance the two feature streams separately, yielding the enhanced features ${\tilde{F}}_{l}$ and ${\tilde{F}}_{h}$ These features are then concatenated along the channel dimension to construct the query feature Q, which contains both shallow- and deep-level information. Subsequently, through Projection operations consisting of convolutional mapping and dimensional reshaping, the two enhanced feature streams are transformed into the key features $K_{x}$ and value features $V_{x}$, respectively. Meanwhile, the two enhanced features are concatenated along the channel dimension and reshaped to construct the query feature. Correlations between the query feature Q and the key feature $K_{x}$ are then calculated, and the corresponding attention weight $A_{x}$ is obtained through Softmax normalization. Afterward, a cross-attention mechanism is employed to achieve bidirectional cross-propagation between the two types of features. Finally, the two cross-enhanced feature streams are concatenated along the channel dimension and fused through convolution to obtain the final output feature $F_{o}$. To describe the cross-fusion process between shallow detail features and deep semantic features in FSFM, the process is divided into four steps: feature mapping, correlation calculation, bidirectional cross-enhancement, and convolutional fusion. The detailed computation is given in Equations (11)–(15).

(11) $$K_{x}=R(\varphi_{x}^{K}({\bar{F}}_{x})),\;x\in \{l,h\},$$

(12) $$V_{x}=R(\varphi_{x}^{V}({\bar{F}}_{x})),\;x\in \{l,h\},$$

(13) $$Q=R(\mathrm{Cat}({\bar{F}}_{l},{\bar{F}}_{h})),$$

(14) $$A_{x}=\mathrm{Softmax}(QK_{x}^{T}),\;x\in \{l,h\},$$

(15) $$F_{o}=\psi (\mathrm{Cat}({\bar{F}}_{l}+R^{-1}(A_{h}V_{h}),{\bar{F}}_{h}+R^{-1}(A_{l}V_{l}))),$$

where $\varphi_{x}^{K}(\cdot )$ and $\varphi_{x}^{V}(\cdot )$ denote the convolutional mapping operations used to generate the key and value features, respectively. R(⋅) denotes the dimensional reshaping operation that converts a spatial feature map into a sequential representation, whereas $R^{-1}(\cdot )$ denotes the inverse reshaping operation that restores the sequential features to a spatial feature map. Cat(⋅) denotes feature concatenation along the channel dimension, the superscript T denotes matrix transposition, and ψ(⋅) denotes the final convolutional fusion operation. In Equation (15), $A_{h}V_{h}$ supplements the shallow branch with deep semantic information, whereas $A_{l}V_{l}$ injects shallow spatial details into the deep branch, thereby achieving bidirectional cross-enhancement between the two feature streams.

The FSFM module can effectively utilize information from different levels to generate high-quality fused feature maps that contain both rich details and deep semantic information, thereby meeting the requirements of insulator small-object defect detection tasks in complex scenarios.

#### 3.2.4. LWSD Lightweight Detection Head

**Figure 6:**
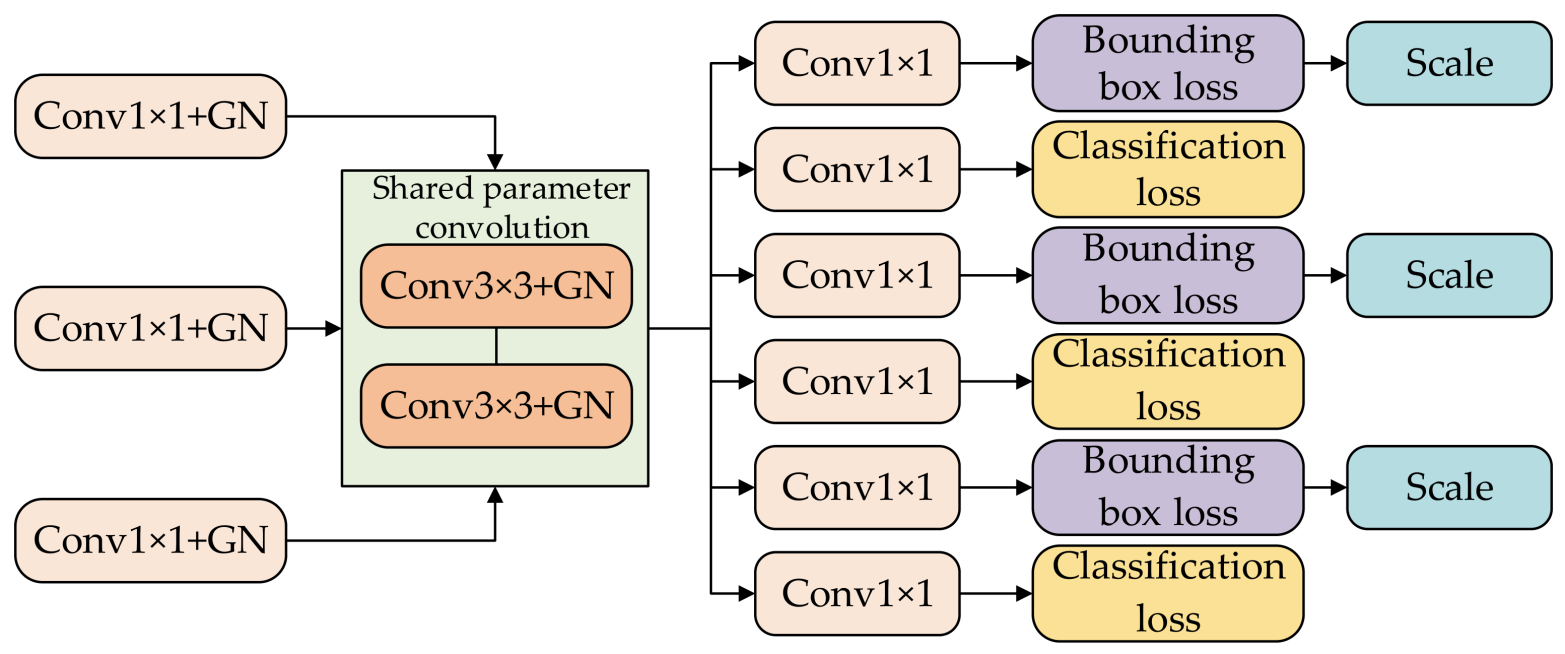
Structure of the LWSD Detection Head.

The original YOLOv8n adopts a decoupled detection head, in which object classification and bounding-box regression are set as two independent branches, enabling the two branches to learn features for different tasks. However, the detection branches at different scales contain independent convolutional structures, resulting in obvious parameter redundancy and repeated computation. This increases the computational burden of the model and is not conducive to the real-time deployment of UAV aerial small-object detection on edge devices. Therefore, this paper introduces a lightweight weight-sharing detection head, LWSD, which uses two 3 × 3 shared convolutional layers to uniformly process features at different scales. In this way, the detection branches at different scales can reuse the same convolutional parameters, thereby reducing the number of parameters and the computational complexity of the detection head. Meanwhile, considering that the statistical results of batch normalization are prone to fluctuations under small-batch training conditions, LWSD adopts group normalization instead of the original batch normalization to improve the stability of feature distributions under different batch sizes. In addition, to address the problem that parameter sharing may lead to inconsistent regression feature distributions at different scales, an independent Scale layer is set in each bounding-box regression branch to calibrate the regression outputs, thereby enhancing the model’s localization ability for aerial small objects at different scales. The structure of the LWSD detection head is shown in Figure 6.

First, the three-scale features output by the FSFM module are fed into independent 1 × 1 convolution and group normalization layers to complete feature channel alignment, ensuring that features at different scales have consistent channel dimensions. Subsequently, the channel-aligned features are sequentially processed by two 3 × 3 convolution and group normalization operations. The parameters of the two 3 × 3 convolutional layers are shared among the three scale detection branches and are used to extract object features with consistent semantic representations. After shared convolution processing, the features at each scale are fed into the bounding-box regression branch and the classification branch, respectively, and bounding-box prediction results and category prediction results are obtained through independent 1 × 1 convolutions. Finally, the bounding-box regression branch uses learnable Scale coefficients to adaptively adjust the regression outputs at different scales, and the bounding-box loss and classification loss are calculated using the ground-truth labels. The feature processing procedure of the LWSD detection head includes channel alignment, shared convolutional feature extraction, scale-calibrated regression, and classification prediction. The specific calculation process is shown in Equations (16)–(19).

(16) $$F_{i}^{\left(0\right)}={\mathrm{GN}}_{i}\left({\mathrm{Conv}}_{1\times 1}^{\left(i\right)}(X_{i})\right),\;i\in \{3,4,5\},$$

(17) $$F_{i}=\delta \left({\mathrm{GN}(\mathrm{Conv}}_{3\times 3}^{2}(\delta ({\mathrm{GN}(\mathrm{Conv}}_{3\times 3}^{1}(F_{i}^{\left(0\right)})))))\right),$$

(18) $$({\hat{B}}_{i},{\hat{C}}_{i})=\left(\alpha_{i}{\mathrm{Conv}}_{1\times 1}^{r(i)}(F_{i}),\;{\mathrm{Conv}}_{1\times 1}^{c(i)}(F_{i})\right),$$

(19) $$L_{\mathrm{LWSD}}=\sum_{i=3}^{5}\left[\lambda_{\mathrm{box}}L_{\mathrm{box}}({\hat{B}}_{i},B_{i})+\lambda_{\mathrm{cls}}L_{\mathrm{cls}}({\hat{C}}_{i},Y_{i})\right],$$

where $X_{i}$ denotes the i-th scale feature output by the FSFM module; $F_{i}^{\left(0\right)}$ denotes the feature after channel alignment through 1 × 1 convolution and group normalization; $F_{i}$ denotes the feature after two shared 3 × 3 convolution layers; GN(⋅) denotes group normalization; δ(⋅) denotes the nonlinear activation function; $\alpha_{i}$ denotes the learnable scale adjustment coefficient; ${\hat{B}}_{i}$ and ${\hat{C}}_{i}$ denote the bounding-box prediction result and category prediction result, respectively; and $B_{i}$ and $Y_{i}$ denote the ground-truth bounding box and ground-truth class label, respectively. Through the above process, LWSD achieves parameter sharing among detection branches at different scales while maintaining the decoupling of classification and localization tasks, and improves the stability and localization accuracy of aerial small-object detection by using scale calibration and group normalization.

In summary, this paper forms the CEF-YOLOv8n method for UAV aerial small-object detection by making targeted improvements to the backbone network, neck network, and detection head of YOLOv8n. Specifically, the CPF module uses partial convolution to reduce redundant computation and enhance the extraction of local details and weak-response features of low-resolution small objects. FGFPN strengthens information interaction among features at different scales through the FFStage feature focusing structure, DySample dynamic upsampling, and SPDConv downsampling, reducing detail loss caused by scale transformation. FSFM uses a cross-attention mechanism to achieve complementary fusion of shallow spatial details and deep semantic information, improving the model’s representation capability for multi-scale and densely distributed targets. LWSD reduces parameter redundancy and computational complexity in the detection head through shared convolution, group normalization, and scale calibration. These modules work collaboratively, enabling CEF-YOLOv8n to enhance small-object feature extraction, improve multi-scale information fusion, and increase bounding-box localization capability while maintaining lightweight model design and inference efficiency. Therefore, it provides an effective method for small-object detection and recognition in complex UAV aerial scenarios.

## 4. Experiments and Analysis

The experiments were conducted under the Windows 11 operating system. The hardware platform was configured with a 13th Gen Intel(R) Core(TM) i5-13400 processor at 2.50 GHz, an NVIDIA GeForce RTX 5060 graphics card with 12 GB of video memory, and 32 GB of RAM, the manufacturer is Intel Corporation. The model was built and trained in a Python environment based on the PyTorch 2.0 deep learning framework, and GPU-accelerated computation was implemented using CUDA 11.8. All comparative experiments and ablation experiments were conducted under the same software and hardware environment to ensure the fairness and comparability of the experimental results of different models.

### 4.1. Dataset and Evaluation Metrics

To verify the effectiveness of the proposed CEF-YOLOv8n model in UAV aerial small-object detection, the VisDrone2019 dataset was selected as the source of training and testing data. VisDrone2019 is a commonly used object detection dataset for UAV aerial scenarios. Its images were collected by UAV platforms at different altitudes, from different viewpoints, and under complex background conditions. The dataset contains ten categories of objects, including pedestrians, bicycles, cars, motorcycles, buses, and trucks, with a total of 8599 static images. In this paper, the dataset was divided into training, validation, and test sets at a ratio of 8:1:1 for model training, parameter adjustment, and performance testing. The objects in this dataset exhibit significant scale variations, with a high proportion of small objects. In addition, some scenes contain densely distributed objects, occlusion, and complex backgrounds, which can effectively simulate the problems of low-resolution, large-scale differences, and dense object distribution encountered in small-object detection under UAV aerial environments. Therefore, this dataset is suitable for verifying the recognition and localization capability of the proposed model for aerial small objects.

The experiments adopted mAP50, mAP50-95, the number of parameters (Params), and floating-point operations (FLOPs) as the main evaluation metrics to comprehensively measure the detection accuracy and computational complexity of the model. Among them, mAP denotes mean average precision, which is used to evaluate the overall detection performance of the model in multi-class object detection tasks. mAP50 denotes the mean average precision of all categories when the Intersection over Union (IoU) threshold is 0.5, mainly reflecting the detection capability of the model under relatively relaxed localization conditions. mAP50-95 denotes the average detection accuracy when the IoU threshold varies from 0.5 to 0.95 at an interval of 0.05, which can more comprehensively reflect the overall performance of the model under different localization accuracy requirements. Params denotes the total number of trainable parameters in the model, mainly reflecting the spatial complexity and storage cost of the model. FLOPs denotes the number of floating-point operations required for one forward inference of the model and is used to measure the computational complexity and inference cost of the model. Through the above metrics, the performance of CEF-YOLOv8n can be comprehensively evaluated from three aspects: detection accuracy, model size, and computational efficiency.

### 4.2. Comparative Experiments

#### 4.2.1. Comparative Experiment on the CPF Module

To verify the effectiveness of the CPF module in feature extraction for UAV aerial small objects, this paper keeps the neck network and detection head unchanged and selects several common lightweight or attention-enhanced feature extraction structures for comparison with the CPF module, including GhostC2f, RepC2f, C2f-CA, and C2f-EMA. These modules improve the backbone network from different perspectives, such as reducing redundant convolutional computation, enhancing re-parameterized feature representation, introducing coordinate attention, and modeling multi-scale attention. In this paper, these modules are used to replace some C2f modules in the original YOLOv8n backbone network, and comparative experiments are conducted under the same training parameters and dataset partition conditions. The experimental results are shown in Table 1.

As shown in Table 1, all the evaluated backbone feature extraction modules improve the detection performance of YOLOv8n to some extent, although their detection accuracy and model complexity differ. GhostC2f reduces the number of parameters and FLOPs to 2.62 M and 7.2 G, respectively, but improves mAP50 and mAP50-95 by only 0.3 and 0.2 percentage points over the baseline model. This indicates that, although GhostC2f reduces computational cost, its ability to enhance the features of low-resolution small objects is relatively limited. RepC2f strengthens convolutional feature representation through a re-parameterized structure, improving the two-accuracy metrics by 0.5 and 0.3 percentage points, respectively. C2f-CA and C2f-EMA employ attention mechanisms to emphasize features in key regions, achieving mAP50 values of 34.8% and 34.9%, respectively. However, both their parameter counts and FLOPs are higher than those of the baseline model, indicating that attention computation introduces additional overhead while enhancing object responses.

In comparison, after CPF is adopted, the model achieves an mAP50 of 35.1% and an mAP50-95 of 20.6%, representing improvements of 0.9 and 0.6 percentage points over the baseline model, respectively. Meanwhile, the number of parameters decreases from 3.2 M to 2.78 M, and FLOPs decrease from 8.7 G to 7.9 G. CPF processes the input features through a partial convolution branch and a feature-preservation branch in parallel. The partial convolution branch extracts the local spatial information of small objects, whereas the feature-preservation branch reduces the loss of original details during successive convolution operations. The shortcut connection and subsequent feature fusion further strengthen information propagation across different feature levels. By applying spatial convolution to only a subset of the channels while retaining the original features of the remaining channels, CPF reduces the redundant computation caused by full-channel convolution while preserving the details and semantic representations of low-resolution small objects. Therefore, compared with the other lightweight or attention-enhanced modules, CPF improves detection accuracy while maintaining a relatively low parameter count and computational cost.

#### 4.2.2. Comparative Experiment on Neck Networks

To verify the effectiveness of the FGFPN neck network in multi-scale feature fusion, this paper keeps the backbone network and detection head unchanged. Gold-YOLO [37], ASF-YOLO [38], DEA-Net [39], MFDS-DETR [40], and FGFPN are used to replace the original YOLOv8n neck network, respectively, and comparisons are conducted under the same experimental conditions. The experimental results are shown in Table 2.

Table 2 presents the effects of different neck networks on multi-scale feature fusion performance. Gold-YOLO, ASF-YOLO, DEA-Net, and MFDS-DETR all improve the mAP50 and mAP50-95 of the model, but their parameter counts and FLOPs also increase accordingly. Among them, DEA-Net achieves an mAP50 of 35.3% and an mAP50-95 of 20.8%, while its parameters and FLOPs increase to 3.55 M and 9.2 G, respectively. In comparison, FGFPN achieves an mAP50 of 35.6% and an mAP50-95 of 21.0%, representing improvements of 1.4 and 1.0 percentage points over the baseline model, respectively. Meanwhile, its parameters and FLOPs are 3.1 M and 8.4 G, both slightly lower than those of the original YOLOv8n. This indicates that its performance improvement does not rely on simply increasing the number of feature channels or enlarging the network scale. In FGFPN, FFStage aggregates features from different levels and strengthens information interaction among P3, P4, and P5, while DySample and SPDConv perform upsampling and downsampling, respectively, during the feature diffusion process to reduce the loss of small-object details caused by scale transformations. These three components are arranged in the order of multi-scale feature aggregation followed by scale diffusion, enabling the aggregated spatial details and contextual information to continue propagating across features of different resolutions and thereby improving the representation capability for objects with significant scale variations. At the same time, DySample and SPDConv avoid the additional computational burden associated with complex sampling structures, allowing FGFPN to improve detection accuracy while maintaining relatively low parameter count and computational cost.

#### 4.2.3. Comparative Experiment on Detection Heads

To analyze the effects of the FSFM feature-semantic fusion module and the LWSD lightweight detection head on model performance, this paper introduces FSFM, LWSD, and their combined structure into the original YOLOv8n, respectively. The experimental results are shown in Table 3.

As shown in Table 3, when FSFM is introduced independently, the mAP50 and mAP50-95 of the model increase from 34.2% and 20.0% to 35.0% and 20.7%, respectively, while the parameters and FLOPs increase to 3.52 M and 9.3 G. FSFM first employs Dense Layers to enhance the corresponding-scale features output by the backbone network and FGFPN. It then establishes interactions between spatial details and contextual semantics through bidirectional cross-attention and fuses the two groups of interacted features. Enhancing the two input feature streams before attention computation helps strengthen the representation of effective information, while bidirectional interaction enables the spatial details retained by the backbone network to complement the contextual semantics aggregated by FGFPN, thereby improving the classification and localization of low-resolution small objects. However, the Dense Layers and cross-attention computation also introduce additional parameters and computational cost.

When LWSD is adopted independently, the mAP50 decreases by only 0.1 percentage points, while the mAP50-95 remains almost unchanged. Meanwhile, the parameters and FLOPs decrease from 3.2 M and 8.7 G to 2.43 M and 6.7 G, respectively. LWSD first uses 1 × 1 convolution and group normalization to align the channels of features at different scales. It then employs a cross-scale shared 3 × 3 convolution to reduce repeated computation across different detection branches and uses independent Scale layers in the bounding-box regression branch to calibrate predictions at different scales. This arrangement ensures that features at different scales have consistent channel dimensions before parameter sharing, while scale calibration preserves the scale adaptability of each detection branch. Therefore, LWSD can substantially reduce model complexity while largely maintaining detection accuracy.

When FSFM and LWSD are used jointly, the model achieves an mAP50 of 35.8% and an mAP50-95 of 21.3%, representing improvements of 1.6 and 1.3 percentage points over the baseline model, respectively. Meanwhile, the parameters and FLOPs decrease to 2.78 M and 7.3 G. FSFM is placed before LWSD so that the detection head first receives multi-scale features integrating spatial details and contextual semantics, after which LWSD compresses redundant computation in the subsequent prediction process through weight sharing. In this way, the enhanced feature representation provided by FSFM partially compensates for the possible information loss caused by weight sharing, while LWSD reduces the additional computational overhead introduced by FSFM. This complementary relationship is the main reason why their joint configuration achieves a better balance between detection accuracy and model complexity.

#### 4.2.4. Comparative Experiment on Generality

To further evaluate the overall performance of CEF-YOLOv8n in UAV aerial small-object detection tasks, RetinaNet, YOLOv5s, YOLOv8n, YOLOv10n, YOLOv11n, YOLOv11s, SUPERLIGHT-DETR [41], BDAD-YOLO [42], and DEPA-YOLO [43] were selected for comparison. These methods cover representative one-stage detectors, YOLO models with different parameter scales, and recently proposed lightweight models designed for small-object detection in UAV and remote-sensing imagery. All comparison models were trained and tested using the same dataset split, input image size, and training settings. The experimental results are presented in Table 4.

As shown in Table 4, the evaluated detection models exhibit different characteristics in terms of detection accuracy and model complexity. RetinaNet and YOLOv5s achieve mAP50 values of 30.1% and 32.6%, respectively, while their parameter counts and FLOPs are substantially higher than those of the lightweight YOLO models. YOLOv8n, YOLOv10n, and YOLOv11n have relatively low model complexity, but their mAP50-95 values are only 20.0%, 20.3%, and 20.7%, respectively, indicating that their overall detection capabilities for low-resolution objects with significant scale variations still have room for improvement. YOLOv11s achieves an mAP50 of 39.0% and an mAP50-95 of 23.5%, but its parameter count and FLOPs also reach 9.4 M and 21.3 G, respectively.

CEF-YOLOv8n achieves an mAP50 of 37.6% and an mAP50-95 of 22.6%, representing improvements of 3.4 and 2.6 percentage points over YOLOv8n, respectively. Meanwhile, its parameter count decreases from 3.2 M to 2.5 M, and its FLOPs decrease from 8.7 G to 6.9 G. Compared with YOLOv11n, the two accuracy metrics are improved by 2.6 and 1.9 percentage points, respectively, while the parameter count is further reduced by 0.1 M. Compared with recently proposed methods specifically designed for small-object detection, the mAP50 of CEF-YOLOv8n is 2.9 and 1.5 percentage points higher than those of SUPERLIGHT-DETR and BDAD-YOLO, respectively, while its mAP50-95 is 1.9 percentage points higher than that of BDAD-YOLO. In addition, CEF-YOLOv8n has a lower parameter count and computational cost. Compared with DEPA-YOLO, CEF-YOLOv8n has an mAP50 that is 0.6 percentage points lower, but its mAP50-95 is 0.2 percentage points higher, while its parameter counts and FLOPs are reduced by 26.5% and 9.2%, respectively. Although the mAP50 and mAP50-95 of CEF-YOLOv8n is 1.4 and 0.9 percentage points lower than those of YOLOv11s, respectively, its parameter count and FLOPs are only 26.6% and 32.4% of those of YOLOv11s.

From the perspective of model architecture, CPF reduces redundant computation in the backbone network by applying convolution to only a subset of the feature channels. FGFPN strengthens multi-scale feature interaction and reduces detail loss during scale transformation through FFStage, DySample, and SPDConv. FSFM supplements the spatial details and category semantics of small objects through bidirectional cross-attention, while LWSD reduces repeated computation in the detection head through cross-scale weight sharing. Among these modules, FGFPN and FSFM mainly improve small-object feature representation and fusion quality, whereas CPF and LWSD reduce the parameter count and computational cost while retaining effective features. Through the joint effects of these modules, CEF-YOLOv8n achieves a substantial improvement in detection accuracy while reducing model complexity, demonstrating a favorable balance between detection performance and computational complexity.

### 4.3. Ablation Experiments

To verify the effectiveness of each improved module proposed in this paper, YOLOv8n is used as the baseline model. Under the same training parameters and dataset split conditions, the CPF feature extraction module, FGFPN feature pyramid network, FSFM feature-semantic fusion module, and LWSD lightweight detection head are introduced, respectively, and further ablation experiments are conducted on different module combinations. The experiments adopt mAP50, mAP50-95, Params, and FLOPs as evaluation metrics to comprehensively analyze the effects of each module on detection accuracy, the number of model parameters, and computational complexity. The experimental results are shown in Table 5.

The ablation results show that each module exhibits distinct performance characteristics when introduced independently. After incorporating CPF, the model’s mAP50 and mAP50-95 increase from 34.2% and 20.2% to 35.2% and 20.7%, respectively, while the parameter counts and FLOPs decrease from 3.2 M and 8.7 G to 2.8 M and 7.9 G. These results indicate that the partial convolution and feature-preservation structures improve the local feature representation of low-resolution small objects while reducing redundant computation. FGFPN increases the mAP50 and mAP50-95 to 35.7% and 21.2%, respectively, achieving a relatively substantial accuracy improvement among the individual modules. Meanwhile, the parameter counts and FLOPs are slightly reduced to 3.1 M and 8.4 G, demonstrating that the multi-scale feature aggregation performed by FFStage and the preservation of information during scale transformation by DySample and SPDConv contribute to improving the feature representation of objects with significant scale variations. FSFM increases the two-accuracy metrics to 35.1% and 20.8%, respectively, but also raises the parameter count and FLOPs to 3.5 M and 9.3 G. This reflects that the Dense Layers, projection mappings, and bidirectional cross-attention enhance the complementarity between spatial details and category semantics while introducing additional computational overhead. LWSD reduces the parameter count and FLOPs to 2.4 M and 6.7 G, respectively, while largely maintaining detection accuracy. This indicates that cross-scale weight sharing reduces repeated parameters across detection branches, whereas group normalization and the Scale layers help preserve prediction stability for features at different scales.

The results obtained with different two-module combinations further demonstrate the interactions among the proposed structures. When CPF and FGFPN are combined, the mAP50 and mAP50-95 reach 36.6% and 21.7%, respectively, indicating that the local details retained in the backbone network can continue to participate in multi-scale feature aggregation within the neck network. The combination of CPF and FSFM achieves mAP50 and mAP50-95 values of 36.1% and 21.3%, respectively, demonstrating that local information preservation provides a sufficient detail foundation for the subsequent fusion of shallow and deep features. The combination of FGFPN and FSFM achieves an mAP50 of 36.6% and an mAP50-95 of 21.8%, indicating that further fusion of the aggregated multi-scale features with the spatial information from the backbone network improves object classification and localization performance, although the parameter count increases to 3.6 M.

Among the two-module combinations containing LWSD, CPF + LWSD has a parameter count of 2.0 M and FLOPs of 5.9 G, making it the configuration with the lowest complexity among all two-module combinations, while still achieving an mAP50 of 35.3% and an mAP50-95 of 20.8%. Both FGFPN + LWSD and FSFM + LWSD achieve mAP50 and mAP50-95 values of 35.8% and 21.3%, respectively. Their parameter counts are 2.6 M and 2.8 M, while their FLOPs are 7.1 G and 7.3 G, respectively. These results indicate that placing LWSD after the feature extraction or fusion structures can reduce repeated computation in the detection head while preserving, to a certain extent, the feature-enhancement effects obtained by the preceding modules.

When CPF, FGFPN, and FSFM are combined, the model’s mAP50 and mAP50-95 further increase to 37.3% and 22.3%, respectively. This demonstrates that the small-object details retained by the backbone network can form more comprehensive feature representations after multi-scale aggregation and subsequent fusion with spatial information at the corresponding scales. In comparison, the combination of CPF, FGFPN, and LWSD has a parameter count of 2.2 M and FLOPs of 6.3 G, while still achieving an mAP50 of 36.7% and an mAP50-95 of 21.8%, highlighting the advantage of this configuration in model compression. The two three-module configurations emphasize detection accuracy and computational complexity, respectively, providing a basis for the joint incorporation of FSFM and LWSD into the complete model.

When all four modules are incorporated, CEF-YOLOv8n achieves an mAP50 of 37.7% and an mAP50-95 of 22.7%, with a parameter count of 2.6 M and FLOPs of 6.9 G. Compared with the CPF + FGFPN + FSFM combination, the addition of LWSD substantially reduces model complexity while maintaining detection accuracy. Compared with the CPF + FGFPN + LWSD combination, the addition of FSFM further improves both accuracy metrics. Overall, CPF and FGFPN improve local-detail extraction and multi-scale information interaction, respectively; FSFM supplements the spatial information and category semantics across different feature levels; and LWSD controls the parameter count and computational overhead of the detection head. By performing complementary functions at different stages of feature processing, the four modules enable the complete model to achieve a favorable balance between detection accuracy and computational complexity.

### 4.4. Visualization Experiments

#### 4.4.1. Model Performance Analysis

**Figure 7:**
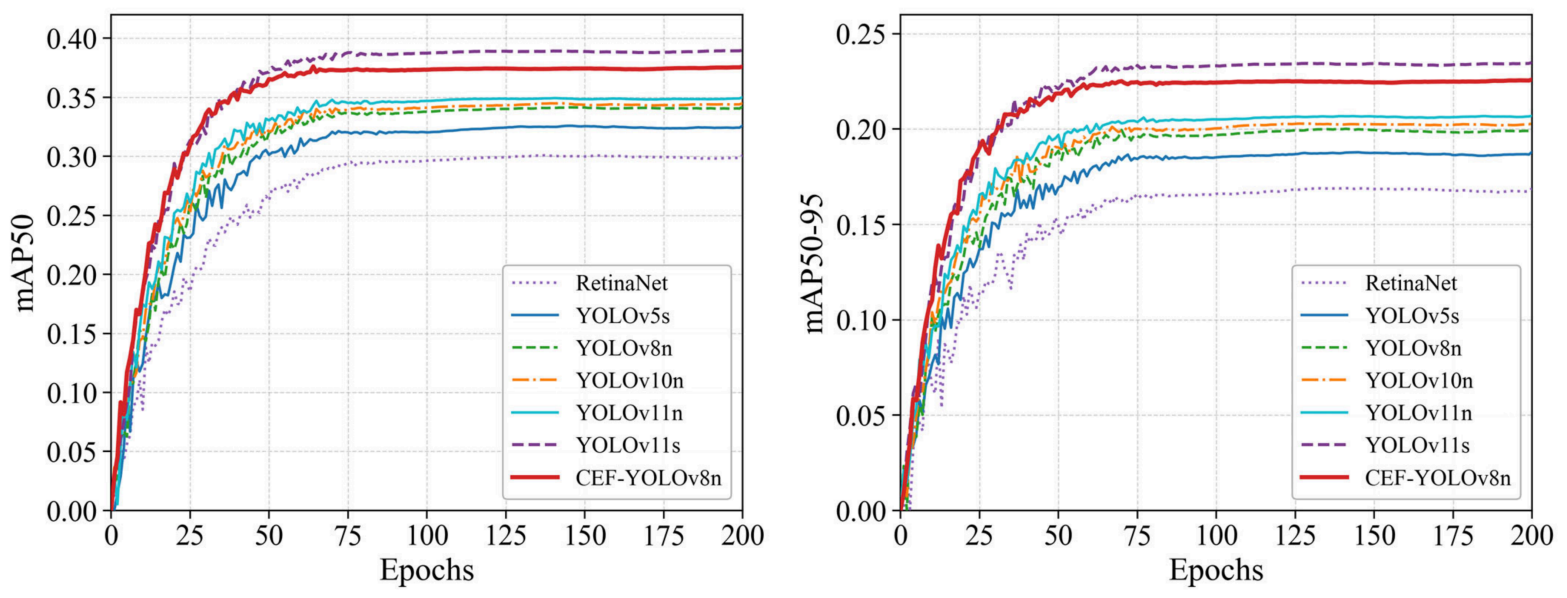
Comparison of mAP50 and mAP50-95 Curves.

To further analyze the convergence characteristics and changes in detection performance of the CEF-YOLOv8n model during training, this paper plots the mAP50 and mAP50-95 curves of each model during the training process, as shown in Figure 7. Among them, mAP50 is used to measure the overall object detection capability of the model when the IoU threshold is 0.5, while mAP50-95 comprehensively considers the average detection accuracy under multiple IoU thresholds and can more strictly reflect the bounding-box localization quality and overall detection performance of the model. By comparing the curve changes of the two models at different training epochs, the effects of improved structures such as CPF, FGFPN, FSFM, and LWSD on model convergence speed, detection accuracy, and training stability can be intuitively evaluated.

As can be seen from the comparison of the mAP50 and mAP50-95 curves, the mAP50 and mAP50-95 of each detection model increase rapidly in the early stage of training and gradually stabilize after approximately 70–80 epochs. This indicates that each model can effectively learn target features in UAV aerial images after a certain number of training epochs. The proposed CEF-YOLOv8n outperforms RetinaNet, YOLOv5s, YOLOv8n, YOLOv10n, and YOLOv11n in both metrics, and its final mAP50 and mAP50-95 stabilize at around 37.68% and 22.67%, respectively, only slightly lower than those of YOLOv11s, which has a larger number of parameters. Meanwhile, the CEF-YOLOv8n curve shows smaller fluctuations in the middle and late stages of training, demonstrating good convergence stability. This result indicates that the collaborative effect of the CPF feature extraction module, FGFPN neck network, FSFM feature-semantic fusion module, and LWSD lightweight detection head can enhance the model’s representation capability for detailed features of small targets in aerial images and multi-scale semantic information. According to the overall trend of the curves, CEF-YOLOv8n achieves high detection accuracy and bounding-box localization performance while maintaining a relatively stable training process.

#### 4.4.2. Confusion Matrix Analysis

**Figure 8:**
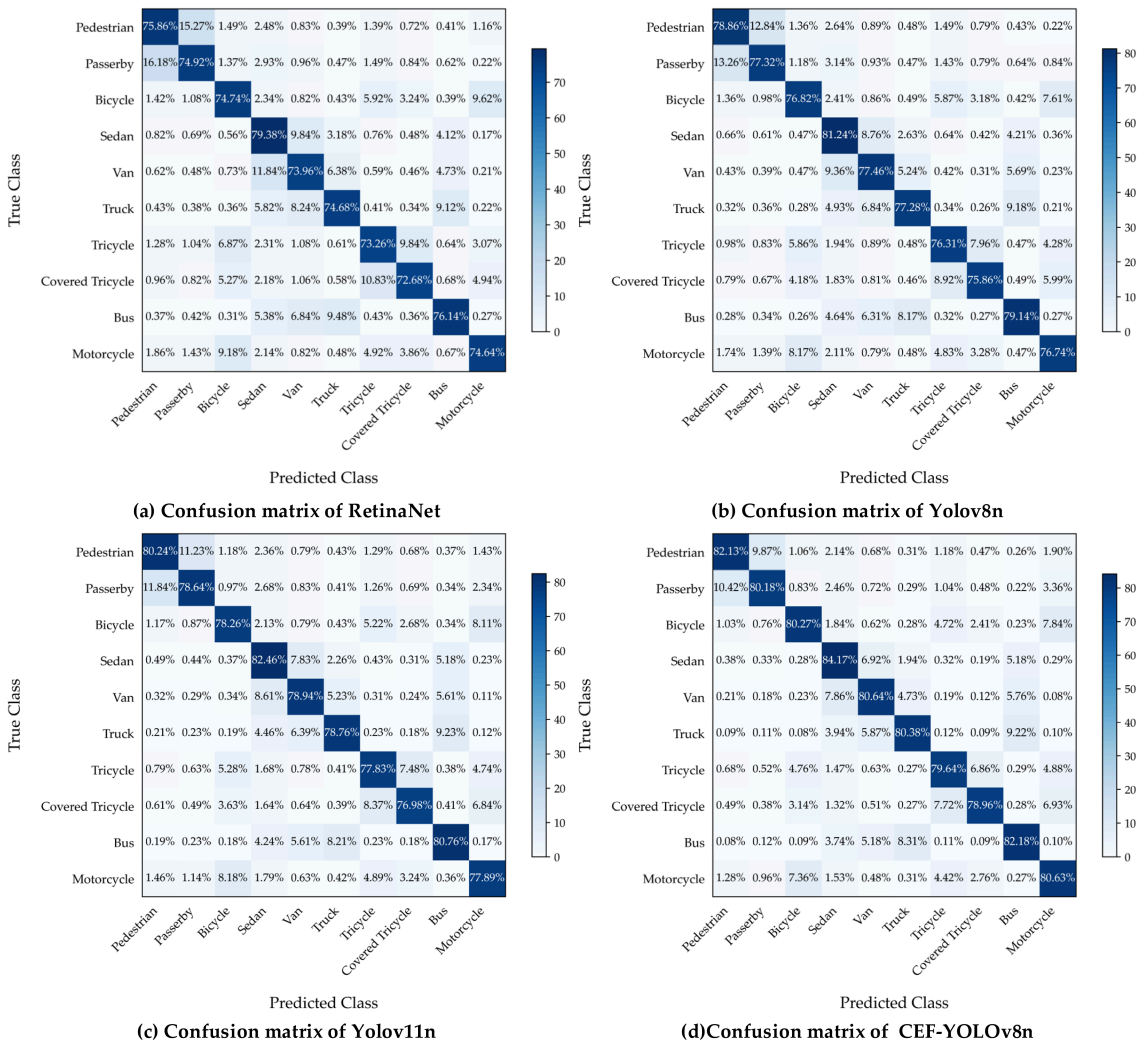
Confusion Matrix.

To further analyze the recognition capability of different models for each target category and the confusion relationships among categories, the confusion matrices of RetinaNet, YOLOv8n, YOLOv11n, and the proposed CEF-YOLOv8n for the ten target categories are plotted based on the VisDrone2019 dataset. The results are shown in Figure 8.

As shown in Figure 8, the recognition results of the four models for all categories are mainly concentrated along the main diagonal, indicating that each model possesses a certain degree of inter-class discrimination capability. However, clear differences can be observed in the diagonal values and off-diagonal misclassification rates. In Figure 8a, the average diagonal value of RetinaNet across all categories is approximately 75.02%, which is lower than those of the other three models. The proportion of pedestrians misclassified as people is 15.27%, while the proportion of people misclassified as pedestrians is 16.18%. The misclassification rates between tricycles and awning tricycles are 9.84% and 10.83%, respectively, indicating that RetinaNet remains insufficiently capable of distinguishing low-resolution objects with similar appearances. In Figure 8b, the average diagonal value of YOLOv8n increases to 77.70%. The recognition rates for cars, pedestrians, and buses reach 81.24%, 78.86%, and 79.14%, respectively. Compared with RetinaNet, the misclassification rates between pedestrians and people and between tricycles and awning tricycles are reduced. Nevertheless, relatively evident category confusion remains between bicycles and motorcycles and between trucks and buses. In Figure 8c, the average diagonal value of YOLOv11n further increases to 79.08%. The recognition rates for cars and buses reach 82.46% and 80.76%, respectively, and the recognition results for most categories are superior to those of YOLOv8n. Meanwhile, the bidirectional misclassification rates between pedestrians and people decrease to 11.23% and 11.84%, respectively. However, a certain degree of misclassification remains for visually similar objects, such as tricycles and awning tricycles. These results demonstrate that YOLOv8n and YOLOv11n exhibit stronger overall category discrimination capabilities than RetinaNet in UAV aerial small-object detection tasks, although further improvement is still required when objects are small, densely distributed, or morphologically similar.

Compared with the above models, the recognition results of CEF-YOLOv8n in Figure 8d are more concentrated along the main diagonal. Its average diagonal value reaches 80.92%, representing improvements of 5.90, 3.22, and 1.84 percentage points over RetinaNet, YOLOv8n, and YOLOv11n, respectively. The car category achieves the highest recognition rate of 84.17%. The recognition rates for pedestrians, people, bicycles, vans, trucks, buses, and motorcycles are all approximately 80% or higher, indicating that the proposed method can effectively extract the local details and category-specific features of vehicles, pedestrians, and non-motorized objects. For easily confused categories, the recognition rates of tricycles and awning tricycles reach 79.64% and 78.96%, respectively, which are 1.81 and 1.98 percentage points higher than those of YOLOv11n. The proportion of tricycles misclassified as awning tricycles decreases from 7.48% to 6.86%, while the proportion of awning tricycles misclassified as tricycles decreases from 8.37% to 7.72%. In addition, the proportions of pedestrians misclassified as people and people misclassified as pedestrians decrease to 9.87% and 10.42%, respectively, while the confusion between bicycles and motorcycles is also alleviated. These improvements are mainly attributed to CPF, which preserves the local details of low-resolution small objects; FGFPN, which aggregates features at different scales; and FSFM, which complementarily fuses spatial details with category semantics. Together, these modules improve the model’s ability to distinguish densely distributed and morphologically similar objects. Nevertheless, some confusion remains between trucks and buses, indicating that the model’s ability to discriminate vehicle objects with similar structural characteristics can be further improved.

#### 4.4.3. Detection Performance Analysis

**Figure 9:**
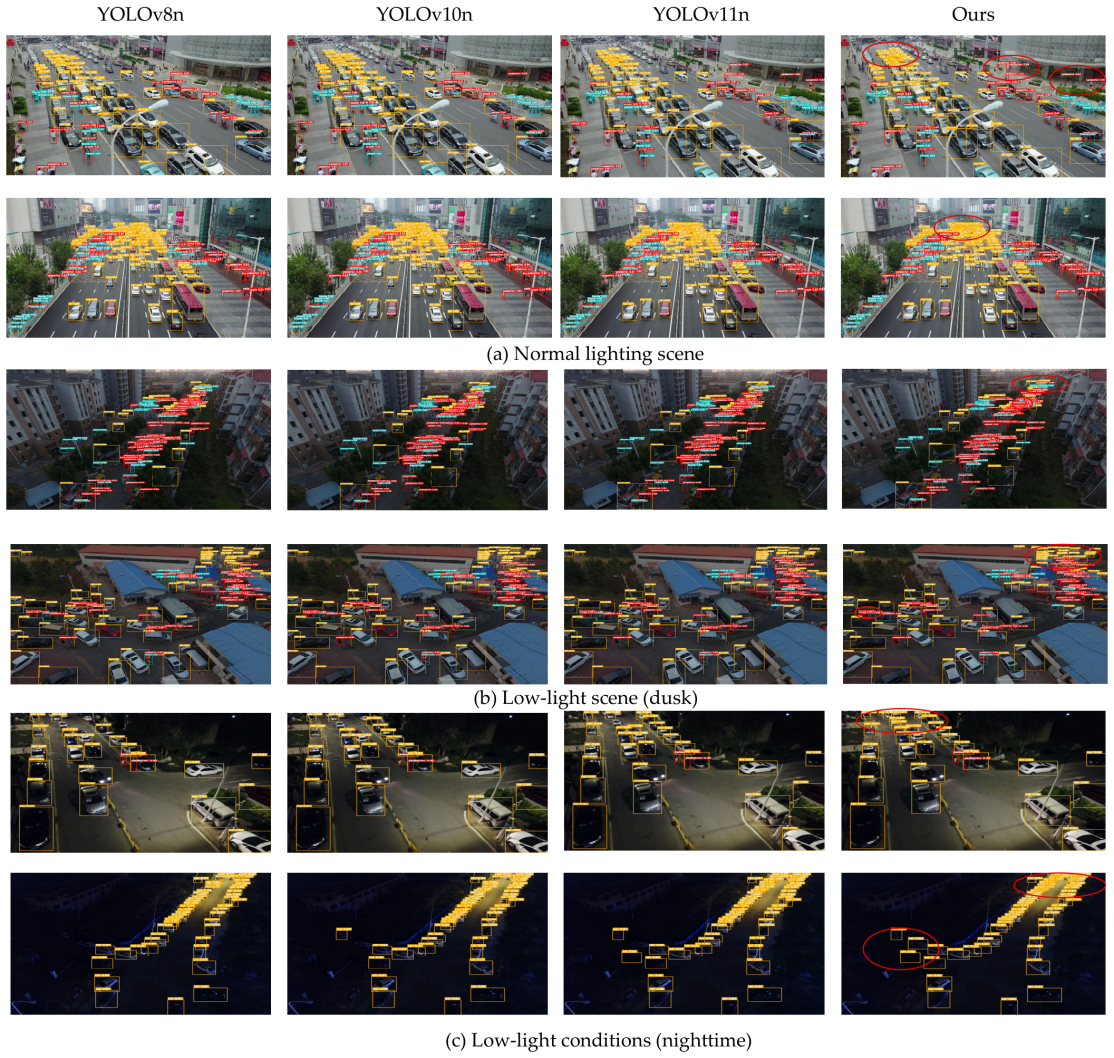
Comparison of Detection Results in Different Scenarios.

To further analyze the detection performance of CEF-YOLOv8n in different UAV aerial scenarios, three representative types of scenes—normal illumination, low-light dusk, and low-light nighttime—were selected from the VisDrone2019 test set for visual comparison. Each type contains two aerial images with different object-distribution characteristics, covering detection challenges such as distant small objects, densely distributed objects, partial occlusion, background interference, and low object-to-background contrast. Figure 9 presents the detection results of YOLOv8n, YOLOv10n, YOLOv11n, and CEF-YOLOv8n from left to right, with red ellipses indicating regions where notable differences exist among the detection results of the models.

In the normal-illumination scenes shown in Figure 9a, the aerial images exhibit relatively high visibility, but objects at the far end of the road occupy only a small number of pixels, and vehicles and pedestrians are densely distributed. In the first image, YOLOv8n, YOLOv10n, and YOLOv11n fail to completely detect some continuously distributed car objects at the far end of the road. They also exhibit varying degrees of missed detection for several pedestrian objects near the entrance of the building and the adjacent sidewalk on the right side. In contrast, CEF-YOLOv8n identifies more distant car and pedestrian objects within the red-circled regions and generates relatively independent bounding boxes for adjacent objects. In the second image, a large number of small and closely spaced vehicles are concentrated at the far end of the commercial road. As the imaging distance increases, the edge and appearance information of these objects gradually weakens. All three comparison models exhibit some missed detections in the distant dense region, whereas CEF-YOLOv8n produces relatively continuous car detection results within the red-circled region. These differences under normal illumination mainly occur in regions containing distant, small-scale, and densely distributed objects. CPF preserves more edge and texture information of small objects in the backbone network, thereby providing sufficient local features for subsequent feature fusion. FGFPN strengthens information interaction among the P3, P4, and P5 levels, allowing the local responses of distant small objects to continue propagating across features at different scales. FSFM further fuses the spatial information retained by the backbone network with the contextual semantics aggregated by FGFPN, which helps distinguish closely arranged vehicles and pedestrians near buildings. Therefore, CEF-YOLOv8n produces more complete detection results for distant and densely distributed small objects under normal illumination.

Figure 9b presents the detection results under low-light dusk conditions. In the first aerial image of a residential area, extensive shadows cast by buildings and trees reduce the contrast between objects and the background, and some car, pedestrian, and bicycle objects retain only weak contour information. YOLOv8n, YOLOv10n, and YOLOv11n can identify relatively clear objects in the middle of the road, but some objects at the far end of the road and in areas covered by tree shadows remain undetected. CEF-YOLOv8n produces more complete detection results for vehicles, pedestrians, and non-motorized objects in the red-circled region at the top of the image. In the second image of a parking area, vehicles, pedestrians, and non-motorized objects are densely distributed around buildings, while some objects are partially occluded by vehicles and building facilities. The three comparison models fail to sufficiently detect some pedestrian objects in the shadowed region on the left and small objects in the dense region at the upper right of the image, whereas CEF-YOLOv8n generates more valid bounding boxes in the corresponding regions. Under dusk conditions, reduced illumination further weakens the edge, texture, and color differences of low-resolution objects. CPF performs spatial convolution on only a subset of channels while preserving the original information in the remaining channels, helping alleviate the attenuation of weak object responses caused by successive convolution operations. FGFPN exploits complementary information among features at different scales, improving scale adaptability when distant and nearby objects coexist. FSFM supplements the local information of objects in shadowed and occluded regions with contextual semantics, thereby reducing feature confusion between densely distributed objects and complex backgrounds. These three modules improve object representation in dusk scenes from the perspectives of local-information preservation, multi-scale information interaction, and shallow–deep feature fusion, respectively, enabling CEF-YOLOv8n to exhibit visibly fewer missed detections in the red-circled regions.

Figure 9c further compares the detection results of different models under low-light nighttime conditions. In the first image, vehicles are mainly illuminated by streetlights or headlights, and the object textures and vehicle contours in dark regions are incomplete. YOLOv8n, YOLOv10n, and YOLOv11n can detect nearby vehicles with sufficient illumination, but their detection of some low-brightness, small-scale car objects at the far end of the road is incomplete. In contrast, CEF-YOLOv8n produces more continuous detection results in the red-circled region at the top of the image. In the second image, the UAV flies at a relatively high altitude, and the overall image brightness is low. Some vehicles on the left side of the road exhibit only faint contours. The three comparison models show varying degrees of missed detection for these vehicles in dark regions. In addition to identifying the continuously distributed vehicles in the middle and upper parts of the road, CEF-YOLOv8n also detects several low-contrast car objects in the red-circled region on the left. In nighttime scenes, the visible features of vehicles are mainly concentrated in local edges and a small number of highlighted regions. CPF preserves weak edge and local texture information, increasing the likelihood that vehicles in dark regions generate effective feature responses. FGFPN reduces the loss of positional and edge information of small objects during feature upsampling and downsampling, enabling vehicle features at different distances to be utilized across multiple scales. FSFM further enhances the distinction between objects in dark regions and the road background by incorporating contextual semantic information. These effects enable CEF-YOLOv8n to achieve more complete detection results for distant and weak-response vehicles under low-light nighttime conditions.

### 4.5. Model Deployment Experiments

To directly and objectively evaluate the effectiveness of the proposed algorithm on embedded platforms, inference tests were conducted on the VisDrone2019 test set using YOLOv8n as the comparison model. Two embedded platforms, Jetson Orin Nano Super and Jetson AGX Orin, were employed. Jetson Orin Nano Super was used to evaluate the operating performance of the model on a relatively resource-constrained platform, whereas Jetson AGX Orin was used to analyze its inference efficiency on a high-performance edge-computing platform. Three inference modes were adopted: PyTorch-FP32 without TensorRT acceleration, TensorRT single-precision floating-point inference (TensorRT-FP32), and TensorRT half-precision floating-point inference (TensorRT-FP16). These experiments were used to analyze the effects of different inference optimization strategies on detection accuracy and inference speed. In all experiments, the batch size was set to 1, the input image size was uniformly resized to 640 × 640, and the same model weights, test data, and evaluation criteria were used. The main specifications of the embedded platforms are listed in Table 6. The comparative results on Jetson Orin Nano Super and Jetson AGX Orin are presented in Table 7 and Table 8, respectively.

During deployment, the network architectures and trained weights of YOLOv8n and CEF-YOLOv8n were first exported to the ONNX format. TensorRT 10.3 was then used to build inference engines on the Jetson Orin Nano Super and Jetson AGX Orin platforms. Three inference modes were evaluated: PyTorch-FP32, TensorRT-FP32, and TensorRT-FP16. PyTorch-FP32 directly performs single-precision inference using the original PyTorch model and serves as the performance baseline without TensorRT optimization. TensorRT-FP32 performs single-precision computation and optimizes the inference process through layer fusion, computational kernel selection, and memory reuse. TensorRT-FP16 allows network layers that support half-precision computation to be executed in FP16, thereby reducing computational and storage overhead while largely maintaining detection accuracy.

As shown in Table 7, TensorRT optimization substantially improves the inference speeds of both models on the Jetson Orin Nano Super platform. When CEF-YOLOv8n is evaluated using PyTorch-FP32, TensorRT-FP32, and TensorRT-FP16, its inference speeds are 25.7, 48.4, and 68.1 FPS, respectively, with corresponding average latencies of 38.91, 20.66, and 14.68 ms. Compared with PyTorch-FP32, TensorRT-FP32 and TensorRT-FP16 improve the inference speed by 88.3% and 165.0%, respectively, while reducing peak memory usage from 2980 MB to 1680 MB and 1280 MB. These results indicate that TensorRT computational graph optimization and half-precision computation can effectively reduce inference overhead on resource-constrained platforms. Under the three inference modes, the mAP50 of CEF-YOLOv8n remains within 37.6–37.7%, while its mAP50-95 remains within 22.6–22.7%, indicating that TensorRT optimization and FP16 computation do not noticeably affect detection accuracy. Under TensorRT-FP16, CEF-YOLOv8n improves mAP50 and mAP50-95 by 3.4 and 2.5 percentage points over YOLOv8n, respectively. Its inference speed increases from 61.6 to 68.1 FPS, corresponding to an improvement of 10.6%, while its average latency decreases from 16.23 to 14.68 ms.

In terms of resource consumption, CEF-YOLOv8n has a peak memory usage of 1280 MB under TensorRT-FP16, accounting for approximately 15.6% of the 8 GB unified memory available on the Orin Nano Super. This leaves sufficient memory for the operating system, image buffering, and other tasks. Its average input power consumption is 18.3 W, which is also slightly lower than the 18.8 W of YOLOv8n. These results demonstrate that CEF-YOLOv8n improves detection accuracy and inference speed without increasing platform memory usage or input power consumption, confirming its feasibility for deployment on embedded platforms with relatively limited computational resources.

As shown in Table 8, the Jetson AGX Orin provides high inference performance for object detection models under the 30 W power mode. When CEF-YOLOv8n is evaluated using PyTorch-FP32, TensorRT-FP32, and TensorRT-FP16, its inference speeds are 46.8, 89.9, and 129.2 FPS, respectively, with corresponding average latencies of 21.37, 11.12, and 7.74 ms. Compared with PyTorch-FP32, TensorRT-FP32 and TensorRT-FP16 improve inference speed by 92.1% and 176.1%, respectively, while reducing peak memory usage from 3330 MB to 1840 MB and 1400 MB. Under the three inference modes, the mAP50 of CEF-YOLOv8n remains within 37.6–37.7%, while its mAP50-95 remains within 22.6–22.7%, indicating that detection accuracy remains essentially stable. Under TensorRT-FP16, CEF-YOLOv8n achieves a 10.6% higher FPS than YOLOv8n and reduces the average latency from 8.56 to 7.74 ms, while improving mAP50 and mAP50-95 by 3.4 and 2.5 percentage points, respectively.

Regarding resource consumption, CEF-YOLOv8n has a peak memory usage of 1400 MB under TensorRT-FP16, accounting for approximately 2.1% of the 64 GB unified memory available on the AGX Orin, with an average input power consumption of 24.2 W. Compared with YOLOv8n under the same inference mode, CEF-YOLOv8n reduces peak memory usage from 1460 to 1400 MB and average input power consumption from 24.8 to 24.2 W. These results indicate that CEF-YOLOv8n maintains low inference latency and resource consumption on a high-performance embedded platform.

Considering the experimental results on both platforms, TensorRT optimization improves model inference speed while largely maintaining detection accuracy and reducing inference latency and memory usage. Under TensorRT-FP16, CEF-YOLOv8n achieves 68.1 FPS on the Jetson Orin Nano Super and 129.2 FPS on the Jetson AGX Orin, with average latencies of 14.68 and 7.74 ms, respectively. Both values are below the 33.33 ms latency corresponding to real-time processing at 30 FPS. Peak memory usage on the two platforms is 1280 and 1400 MB, respectively, both within the available memory capacities of the corresponding platforms. The average input power consumptions are 18.3 and 24.2 W, respectively, neither exceeding the configured power budgets of 25 and 30 W. The experiments on the Jetson Orin Nano Super reflect the operating performance of the model under relatively constrained computational and power conditions, whereas those on the Jetson AGX Orin evaluate its inference efficiency on a high-performance edge-computing platform. Compared with YOLOv8n under the same platform and inference mode, CEF-YOLOv8n achieves higher detection accuracy and inference speed without increasing peak memory usage or average input power consumption. These results demonstrate that the proposed model achieves a favorable balance between detection performance and operating efficiency and is feasible for embedded deployment under different computational-resource conditions.

## 5. Conclusions

To address the detection challenges caused by the high proportion, low resolution, substantial scale variation, and dense distribution of small objects in UAV aerial images, this study proposes CEF-YOLOv8n, a small-object detection model developed on the basis of YOLOv8n. CPF, FGFPN, FSFM, and LWSD are incorporated into the backbone, neck, feature-fusion stage, and detection head, respectively, allowing the local details, multi-scale information, and contextual semantics of low-resolution small objects to be effectively utilized throughout the network while controlling model complexity through a lightweight detection structure. Experimental results on the VisDrone2019 dataset show that CEF-YOLOv8n achieves an mAP50 of 37.6% and an mAP50-95 of 22.6%, representing improvements of 3.4 and 2.6 percentage points over YOLOv8n, respectively. Meanwhile, Params decreases from 3.2 M to 2.5 M, and FLOPs decreases from 8.7 G to 6.9 G. The ablation and comparative experiments demonstrate that the proposed modules play complementary roles in feature representation, cross-scale fusion, and model compression, enabling the model to achieve a favorable balance between detection accuracy and computational complexity. Under TensorRT-FP16 inference, CEF-YOLOv8n achieves 68.1 and 129.2 FPS on the Jetson Orin Nano Super and Jetson AGX Orin platforms, respectively, with average latencies of 14.68 and 7.74 ms, further demonstrating the feasibility of embedded deployment under different computational-resource conditions. The current experiments are primarily conducted on the VisDrone2019 dataset, and performance variations across different object-size ranges have not yet been evaluated separately. The generalization capability of the model across different datasets and under extremely low illumination, severe occlusion, and ultra-dense target conditions requires further validation. In addition, the current embedded deployment experiments mainly focus on inference performance under static testing conditions, and the variations in detection performance caused by UAV attitude changes and airframe vibration during real-world flight have not yet been quantified. Future work will therefore conduct cross-dataset validation and size-specific object evaluation and will further investigate the detection stability of the model under different flight conditions using real-world UAV video sequences.

## Figures and Tables

**Table 1:** Comparative Experimental Results of Different Backbone Feature Extraction Modules.

Model	mAP50/%	mAP50-95/%	Params/M	FLOPs/G
YOLOv8n	34.2	20.0	3.2	8.7
YOLOv8n + GhostC2f	34.5	20.2	2.62	7.2
YOLOv8n + RepC2f	34.7	20.3	3.08	8.5
YOLOv8n + C2f-CA	34.8	20.4	3.31	8.9
YOLOv8n + C2f-EMA	34.9	20.5	3.36	9.0
YOLOv8n + CPF	35.1	20.6	2.78	7.9

**Table 2:** Comparative Experimental Results of Different Neck Networks.

Model	mAP50/%	mAP50-95/%	Params/M	FLOPs/G
YOLOv8n	34.2	20.0	3.2	8.7
YOLOv8n + Gold-YOLO [37]	35.2	20.7	3.72	9.6
YOLOv8n + ASF-YOLO [38]	35.0	20.5	3.46	9.4
YOLOv8n + DEA-Net [39]	35.3	20.8	3.55	9.2
YOLOv8n + MFDS-DETR [40]	35.1	20.6	3.61	9.1
YOLOv8n + FGFPN	35.6	21.0	3.1	8.4

**Table 3:** Comparative Results of Different Detection Head Structures.

Model	mAP50/%	mAP50-95/%	Params/M	FLOPs/G
YOLOv8n	34.2	20.0	3.2	8.7
YOLOv8n + FSFM	35.0	20.7	3.52	9.3
YOLOv8n + LWSD	34.1	20.0	2.43	6.7
YOLOv8n + FSFM + LWSD	35.8	21.3	2.78	7.3

**Table 4:** Comparative Experimental Results of Different Object Detection Algorithms.

Model	mAP50/%	mAP50-95/%	Params/M	FLOPs/G
RetinaNet	30.1	16.9	36.4	97.8
YOLOv5s	32.6	18.8	7.2	16.5
YOLOv8n	34.2	20.0	3.2	8.7
YOLOv10n	34.5	20.3	2.7	8.2
YOLOv11n	35.0	20.7	2.6	6.3
YOLOv11s	39.0	23.5	9.4	21.3
SUPERLIGHT-DETR [41]	34.7	—	7.5	15.2
BDAD-YOLO [42]	36.1	20.7	3.2	10.8
DEPA-YOLO [43]	38.2	22.4	3.4	7.6
CEF-YOLOv8n	37.6	22.6	2.51	6.9

“—” indicates that the relevant indicator was not provided in the literature that makes a comparison.

**Table 5:** Ablation Experiment Results.

Model	mAP50/%	mAP50-95/%	Params/M	FLOPs/G
YOLOv8n	34.2	20.2	3.2	8.7
YOLOv8n +CPF	35.2	20.7	2.8	7.9
YOLOv8n +FGFPN	35.7	21.2	3.1	8.4
YOLOv8n +FSFM	35.1	20.8	3.5	9.3
YOLOv8n +LWSD	34.1	20.2	2.4	6.7
YOLOv8n + CPF + FGFPN	36.6	21.7	2.7	7.6
YOLOv8n + CPF + FSFM	36.1	21.3	3.1	8.5
YOLOv8n + CPF + LWSD	35.3	20.8	2.0	5.9
YOLOv8n + FGFPN + FSFM	36.6	21.8	3.6	9.0
YOLOv8n + FGFPN + LWSD	35.8	21.3	2.6	7.1
YOLOv8n + FSFM + LWSD	35.8	21.3	2.8	7.3
YOLOv8n + CPF + FGFPN + FSFM	37.3	22.3	3.1	8.2
YOLOv8n + CPF + FGFPN + LWSD	36.7	21.8	2.2	6.3
YOLOv8n + CPF + FGFPN + FSFM + LWSD	37.6	22.6	2.5	6.9

**Table 6:** Specifications and Experimental Environment of the Embedded Platforms.

Item	Embedded Platform Specifications	
Name	Jetson Orin Nano Super Developer Kit	Jetson AGX Orin 64 GB Developer Kit
Memory	8 GB 128-bit LPDDR5	64 GB 256-bit LPDDR5
CPU	6-core Arm Cortex-A78AE 64-bit, 1.7 GHz	12-core Arm Cortex-A78AE 64-bit, 2.2 GHz
GPU	NVIDIA Ampere architecture	
Operating System	Ubuntu 22.04	
Software Environment	Python 3.10, PyTorch 2.5.0, CUDA 12.6	

**Table 7:** Experimental Results on the Jetson Orin Nano Super Platform.

Model	Inference Mode	mAP50/%	mAP50-95/%	FPS	Average Latency/ms	Peak Memory Usage/MB	Average Input Power/W
YOLOv8n	PyTorch-FP32	34.2	20.2	23.4	42.74	3060	22.1
	TensorRT-FP32	34.2	20.2	43.8	22.83	1740	20.2
	TensorRT-FP16	34.2	20.1	61.6	16.23	1330	18.8
CEF-YOLOv8n	PyTorch-FP32	37.7	22.7	25.7	38.91	2980	21.6
	TensorRT-FP32	37.7	22.7	48.4	20.66	1680	19.7
	TensorRT-FP16	37.6	22.6	68.1	14.68	1280	18.3

**Table 8:** Experimental Results on the Jetson AGX Orin Platform.

Model	Inference Mode	mAP50/%	mAP50-95/%	FPS	Average Latency/ms	Peak Memory Usage/MB	Average Input Power/W
YOLOv8n	PyTorch-FP32	34.2	20.2	42.6	23.47	3420	28.7
YOLOv8n	TensorRT-FP32	34.2	20.2	81.4	12.29	1910	26.9
YOLOv8n	TensorRT-FP16	34.2	20.1	116.8	8.56	1460	24.8
CEF-YOLOv8n	PyTorch-FP32	37.7	22.7	46.8	21.37	3330	28.1
CEF-YOLOv8n	TensorRT-FP32	37.7	22.7	89.9	11.12	1840	26.2
CEF-YOLOv8n	TensorRT-FP16	37.6	22.6	129.2	7.74	1400	24.2

## Data Availability

The data that support the findings of this study are available from the corresponding author upon reasonable request.

## References

[1] Shahi T.B., Xu C.Y., Neupane A., Guo W. (2023). Recent advances in crop disease detection using UAV and deep learning techniques. Remote Sens..

[2] Hanzla M., Ali S., Jalal A. (2024). Smart traffic monitoring through drone images via YOLOv5 and Kalman filter. Proceedings of the 2024 5th International Conference on Advancements in Computational Sciences.

[3] Yang X., Zhang Y., Lv W., Wang D. (2021). Image recognition of wind turbine blade damage based on a deep learning model with transfer learning and an ensemble learning classifier. Renew. Energy.

[4] Ren D., Zhang Y., Wang L., Liu X., Chen Y., Zhang H. (2024). FCLG-YOLO: Feature constraint and local guided global feature for fire detection in unmanned aerial vehicle imagery. IEEE J. Sel. Top. Appl. Earth Obs. Remote Sens..

[5] Osco L.P., Marcato J.J., Marques R.A.P., Rodrigues J.A., Eanes A., Li J., Junior J.M., Gonçalves W.N. (2021). A review on deep learning in UAV remote sensing. Int. J. Appl. Earth Obs. Geoinf..

[6] Zhu P., Wen L., Du D., Bian X., Fan H., Hu Q., Ling H. (2022). Detection and tracking meet drones challenge. IEEE Trans. Pattern Anal. Mach. Intell..

[7] Marvasti-Zadeh S.M., Cheng L., Ghanei-Yakhdan H., Kasaei S. (2022). Deep learning for visual tracking: A comprehensive survey. IEEE Trans. Intell. Transp. Syst..

[8] Girshick R., Donahue J., Darrell T., Malik J. (2014). Rich feature hierarchies for accurate object detection and semantic segmentation. Proceedings of the 2014 IEEE Conference on Computer Vision and Pattern Recognition.

[9] Girshick R. (2016). Fast R-CNN. Proceedings of the 2015 IEEE International Conference on Computer Vision.

[10] Ren S.Q., He K.M., Girshick R., Sun J. (2017). Faster R-CNN: Towards real-time object detection with region proposal networks. IEEE Trans. Pattern Anal. Mach. Intell..

[11] Cai Z.W., Vasconcelos N. (2018). Cascade R-CNN: Delving into high quality object detection. Proceedings of the IEEE/CVF Conference on Computer Vision and Pattern Recognition.

[12] Liu W., Anguelov D., Erhan D., Szegedy C., Reed S., Fu C.Y., Berg A.C. (2016). SSD: Single shot MultiBox detector. Proceedings of the 14th European Conference on Computer Vision.

[13] Lin T.Y., Goyal P., Girshick R., He K.M., Dollár P. (2017). Focal loss for dense object detection. Proceedings of the 2017 IEEE International Conference on Computer Vision.

[14] Betti Sorbelli F., Palazzetti L., Pinotti C.M. (2023). YOLO-based detection of Halyomorpha Halys in orchards using RGB cameras and drones. Comput. Electron. Agric..

[15] Zhang X.X., Wang C.Y., Jin J., Huang L. (2023). Object detection of VisDrone by stronger feature extraction Faster R-CNN. J. Electron. Imaging.

[16] Battish N., Kaur D., Chugh M., Poddar S. (2024). SDMNet: Spatially dilated multi-scale network for object detection for drone aerial imagery. Image Vis. Comput..

[17] Wang Z.K., Dong X.M., Xu Y.L. (2025). SCASNet: Spatial context-aware selection network for small object detection in aerial imagery. IEEE J. Sel. Top. Appl. Earth Obs. Remote Sens..

[18] Qiu J.G., Cai F.K., Fu N., Yao Y.F. (2025). YOLO-Air: An efficient deep learning network for small object detection in drone-based imagery. IEEE Access.

[19] Fan B.G., Wang S.Q., Chen K.Y. (2025). Small target detection model for UAV aerial photography based on improved YOLOv8. J. Comput. Appl..

[20] Li X.X., Diao W.H., Mao Y.Q., Gao P., Mao X.H., Li X.M., Sun X. (2023). OGMN: Occlusion guided multi-task network for object detection in UAV images. ISPRS J. Photogramm. Remote Sens..

[21] Yang G.Y., Lei J., Zhu Z.K., Cheng S.Y., Feng Z.L., Liang R.H. (2023). AFPN: Asymptotic feature pyramid network for object detection. Proceedings of the 2023 IEEE International Conference on Systems, Man, and Cybernetics.

[22] Amudhan A.N., Sudheer A.P. (2022). Lightweight and computationally faster hypermetropic convolutional neural network for small size object detection. Image Vis. Comput..

[23] Hu H., Chen S.B., You Z.H., Tang J. (2025). FSENet: Feature suppression and enhancement network for tiny object detection. Pattern Recognit..

[24] Liao D.D., Zhang J.X., Tao Y., Jin X. (2025). ATBHC-YOLO: Aggregate transformer and bidirectional hybrid convolution for small object detection. Complex Intell. Syst..

[25] Du Z.W., Hu Z.J., Zhao G.Y., Jin Y., Ma H.B. (2025). Cross-layer feature pyramid transformer for small object detection in aerial images. IEEE Trans. Geosci. Remote Sens..

[26] Zhang H., Sun W., Sun C.H., He R.F., Zhang Y.M. (2024). HSP-YOLOv8: UAV aerial photography small target detection algorithm. Drones.

[27] Bai Y., Zhou Y.Y., An S.B. (2024). Research on UAV small object detection method improved by YOLOv5. Comput. Eng. Appl..

[28] Fu J.Y., Zhang Z.J., Sun W., Zou K.X. (2024). Improved YOLOv8 small target detection algorithm in aerial images. Comput. Eng. Appl..

[29] Wu M.J., Yun L.J., Chen Z.Q., Zhong T.Z. (2024). Improved YOLOv5s small object detection algorithm in UAV view. Comput. Eng. Appl..

[30] Wang X.H., Hu Y. (2023). UAV image small object detection on complex background. Comput. Eng. Appl..

[31] Zhao X., Chen L.L., Yang W.C., Zhang C.W. (2024). DY-YOLOv5: Target detection for aerial image based on multiple attention. Comput. Eng. Appl..

[32] Chen J.H., Wang X.H. (2024). Dense small object detection algorithm based on improved YOLOv5 in UAV aerial images. Comput. Eng. Appl..

[33] Zhou Q., Tan G.Q., Yin S.L., Li Y.N., Wei D.Q. (2023). Road object detection algorithm based on improved YOLOv5s. Chin. J. Liq. Cryst. Disp..

[34] Liu H.Y., Duan X.H., Lou H.T., Gu J., Chen H.N., Bi L.Y. (2023). Improved GBS-YOLOv5 algorithm based on YOLOv5 applied to UAV intelligent traffic. Sci. Rep..

[35] Zhang H.S., Tan X., Fan M.W., Pan C.S., Xu J., Zhang Y. (2024). Accurate detection method of small-scale vehicles from perspective of unmanned aerial vehicle high-altitude aerial photography. J. Transp. Syst. Eng. Inf. Technol..

[36] Mao G.T., Deng T.M., Yu N.J. (2023). Object detection in UAV images based on multi-scale split attention. Acta Aeronaut. Astronaut. Sin..

[37] Ge Z., Liu S.T., Wang F., Li Z.M., Sun J. (2021). YOLOX: Exceeding YOLO series in 2021. arXiv.

[38] Hou Q.B., Zhou D.Q., Feng J.S. (2021). Coordinate attention for efficient mobile network design. Proceedings of the 2021 IEEE/CVF Conference on Computer Vision and Pattern Recognition.

[39] Wang C.C., He W., Nie Y., Guo J.Y., Liu C.J., Han K., Wang Y.H. (2023). Gold-YOLO: Efficient object detector via gather-and-distribute mechanism. Proceedings of the 37th International Conference on Neural Information Processing Systems.

[40] Kang M., Ting C.M., Ting F.F., Phan R.C.-W. (2024). ASF-YOLO: A novel YOLO model with attentional scale sequence fusion for cell instance segmentation. Image Vis. Comput..

[41] Xu S., Wu Z., Ke Z., Xue Z., Shen M., Xiao W. SUPERLIGHT-DETR: A Lightweight DETR Model for Small Object Detection in Remote Sensing. Proceedings of the 2025 International Conference on Virtual Reality and Visualization (ICVRV).

[42] Sun J.Y., Xu M.J., Zhang J.P., Yan M.X., Cao W., Hou A.L. (2025). Optimized and improved YOLOv8 target detection algorithm from UAV perspective. Comput. Eng. Appl..

[43] Liu C.J., Liu W., Yang W.D., Wang C. (2026). DEPA-YOLO: Drone based small object detection model. J. Front. Comput. Sci. Technol..

